# Genome-wide signals of positive selection in strongylocentrotid sea urchins

**DOI:** 10.1186/s12864-017-3944-7

**Published:** 2017-07-21

**Authors:** Kord M. Kober, Grant H. Pogson

**Affiliations:** 1 Department of Ecology and Evolutionary Biology, University of California, Santa Cruz, USA; 2 Institute for Computational Health Sciences, University of California, San Francisco, USA; 3 Present address: Department of Physiological Nursing, University of California, San Francisco, USA

**Keywords:** Positive selection, Comparative genomics, *Strongylocentrotus purpuratus*, Sea urchins, *d*_N_*/d*_S_ ratios, Pathogens, Sexual conflict

## Abstract

**Background:**

Comparative genomics studies investigating the signals of positive selection among groups of closely related species are still rare and limited in taxonomic breadth. Such studies show great promise in advancing our knowledge about the proportion and the identity of genes experiencing diversifying selection. However, methodological challenges have led to high levels of false positives in past studies. Here, we use the well-annotated genome of the purple sea urchin, *Strongylocentrotus purpuratus*, as a reference to investigate the signals of positive selection at 6520 single-copy orthologs from nine sea urchin species belonging to the family Strongylocentrotidae paying careful attention to minimizing false positives.

**Results:**

We identified 1008 (15.5%) candidate positive selection genes (PSGs). Tests for positive selection along the nine terminal branches of the phylogeny identified 824 genes that showed lineage-specific adaptive diversification (1.67% of branch-sites tests performed). Positively selected codons were not enriched at exon borders or near regions containing missing data, suggesting a limited contribution of false positives caused by alignment or annotation errors. Alignments were validated at 10 loci with re-sequencing using Sanger methods. No differences were observed in the rates of synonymous substitution (*d*_S_), GC content, and codon bias between the candidate PSGs and those not showing positive selection. However, the candidate PSGs had 68% higher rates of nonsynonymous substitution (*d*_N_) and 33% lower levels of heterozygosity, consistent with selective sweeps and opposite to that expected by a relaxation of selective constraint. Although positive selection was identified at reproductive proteins and innate immunity genes, the strongest signals of adaptive diversification were observed at extracellular matrix proteins, cell adhesion molecules, membrane receptors, and ion channels. Many candidate PSGs have been widely implicated as targets of pathogen binding, inactivation, mimicry, or exploitation in other groups (notably mammals).

**Conclusions:**

Our study confirmed the widespread action of positive selection across sea urchin genomes and allowed us to reject the possibility that annotation and alignment errors (including paralogs) were responsible for creating false signals of adaptive molecular divergence. The candidate PSGs identified in our study represent promising targets for future research into the selective agents responsible for their adaptive diversification and their contribution to speciation.

**Electronic supplementary material:**

The online version of this article (doi:10.1186/s12864-017-3944-7) contains supplementary material, which is available to authorized users.

## Background

Understanding the role of natural selection in shaping patterns of genetic variation within and among species has remained a central focus of evolutionary biologists for many decades [1–3]. The recent availability of genome-wide data, coupled with the development of increasingly sophisticated tests for natural selection (reviewed by [4–6]), is finally providing insights into long-standing questions about the prevalence and the targets of positive selection on both protein-coding genes [7, 8] and non-coding regulatory sequences [9, 10]. To date, the most comprehensive studies have been conducted on humans and *Drosophila* where many candidate positively selected genes have been identified and linked with changes in environmental conditions, diet, pathogens, and sexual selection/conflict (see [11–15]). Recent studies combining the historical signals of selection with genome-wide patterns of polymorphism have identified reduced levels of neutral variation in the vicinity of positively selected amino acids or regulatory elements, thus providing compelling evidence for a direct role of diversifying selection (e.g., [10, 16, 17]).

Genome-wide studies of positive selection among closely related species commonly use maximum-likelihood models of codon substitution to detect significantly elevated rates of nonsynonymous substitutions (*d*_N_) relative to synonymous substitutions (*d*_S_) at individual codons across a phylogeny (sites tests) or along specific branches of the gene tree (branch-sites tests) [18]. Although generally conservative, both sites and branch-sites tests have considerable power to detect positive selection provided that the data and alignments are accurate and the sequences are at an appropriate level of divergence [19–21]. However, comparative studies based on a well-annotated reference genome that have used sufficient numbers of species to provide adequate statistical power have been limited primarily to mammals and *Drosophila* [8, 22]. These studies have found that positive selection is more extensive in *Drosophila* than in mammals and genome-wide *d*_N_ */d*_S_ ratios appear to scale inversely with population size (reviewed by [23]. These patterns suggest that species with small evolutionary effective population sizes experience higher substitution rates of mildly deleterious nonsynonymous mutations due to the reduced efficacy of purifying selection. The generality of these findings is unclear and genome-wide tests for positive selection must be expanded to include a wider range of phylogenetic groups possessing a diverse array of ecological and life-history traits.

Previous studies on strongylocentrotid urchins have reported positive selection on a limited number of sperm and egg proteins [24, 25] and genome-wide evidence for adaptive divergence in a deep-water species, *Allocentrotus fragilis* [26]. However, across this broadly-distributed family both the proportion and the identity of genes experiencing positive selection on a genome-wide scale are difficult to predict. On one hand, their large effective population sizes and extensive levels of genetic diversity should lead to an increased effectiveness of natural selection fixing weakly beneficial mutations. However, high levels of gene flow resulting from a highly dispersive planktonic larval phase could act to constrain local adaptation and reduce species-wide divergence. Unlike *Drosophila*, which inhabit a wide range of habitats and differ widely in their ecological associations and life histories [27], most species included in the present study possess broadly overlapping geographic ranges and depth distributions, and are generalists that feed primarily on kelps (see [28]). Sea urchins might thus experience reduced opportunities for positive selection if ecological diversification is as important in the marine environment as it appears to be in terrestrial habitats [29].

A growing body of work has documented that loci functioning in defense or reproduction evolve more rapidly and are more likely to experience positive selection than other genes [4, 30–32]. In broadcast spawners like sea urchins, the limited behavioral interactions between the sexes might weaken the intensity of sexual selection or sexual conflict across the genome [33]. Disease outbreaks are common in sea urchins (e.g., [34, 35]) and in the case of *Diadema antillarium* have impacted large regions such as the entire Caribbean [36]. However, disease outbreaks are often localized and ephemeral, which might limit the spread and fixation of adaptive resistance alleles across a species’ geographic range. The relative importance of sexual selection/conflict or pathogens as selective agents in marine systems is still largely unknown.

The objective of the present study was to investigate the signatures of positive selection across the genomes of nine sea urchins belonging to the family Strongylocentrotidae. Sea urchins represent an excellent group for such tests; they possess high levels of genetic polymorphism [37, 38], large effective population sizes [39], and extensive geographic ranges that are representative of many marine invertebrates and fishes. In combination, these traits should facilitate the fixation of advantageous alleles irrespective of whether they derive from standing variation or from de novo mutation. The genome of the purple sea urchin, *Strongylocentrotus purpuratus*, has been assembled into a high-quality draft with a well-annotated transcriptome and set of gene predictions [40–42]. Our study includes nine of the ten members of the family Strongylocentrotidae that have diverged over the past 3–18 million years [43, 44] representing an appropriate range of evolutionary divergence and adequate taxon sampling to provide sufficient statistical power [19, 45]. Previously, we reported pervasive weak selection acting on synonymous codon usage in *S. purpuratus* [46]. Here, we document extensive signals of positive selection across the genomes of nine strongylocentrotid sea urchins at an unusual assemblage of genes possibly driven by sexual conflict and pathogen evasion.

## Methods

The primary aim of this study is to evaluate the proportion and the identity of genes experiencing diversifying selection using whole-genome sequencing data from nine sea urchin species and the reference genome of *S. purpuratus*. The secondary aim of this study is to test and evaluate the contribution of annotation and alignment errors in generating false positive signals of positive selection that have been identified in previous studies.

### Samples

Samples of eight strongylocentrotid sea urchin species were obtained from the eastern and western North Pacific regions. *Strongylocentrotus droebachiensis* and *S. pallidus* were dredged in 2005 near Friday Harbor, WA as previously described [38]. A sample of *S. franciscanus* was collected by SCUBA in 2005 near Santa Cruz, CA [38]. *Allocentrotus fragilis* was collected in 2006 from a whale fall in Monterey Bay at a depth of 381 m. Samples of *S. nudus* and *S. intermedius* from the eastern coast of South Korea were kindly provided by Y-H Lee in 2006. We obtained samples of *Hemicentrotus pulcherrimus* and *Pseudocentrotus depressus* from Shimoda, Izu Peninusula, Shizuoka Prefecture, Japan from Y. Agatsuma in 2011. Gonad tissues were preserved in 95% EtOH and total DNA extracted as described in [47].

### DNA sequencing

Paired-end reads were obtained for the eight species using the Illumina HiSeq 2000 platforms (Illumina Inc., San Diego, CA). A 6 ng aliquot of genomic DNA from a single individual of each species was fragmented using a BioRuptor (Diagenode Inc., Denville, NJ) using six cycles of 30s on/off at the “High” setting for 5 min. Paired-end libraries were prepared from the fragmented genomic DNA with mean insert sizes of 200–300 bp and sequenced for 100 bp paired-end reads on the Illumina HiSeq 2000 platform by the QB3 Vincent J Coates Genomic Sequencing Laboratory at the University of California, Berkeley (QB3 VJC GSL; http://qb3.berkeley.edu/gsl/Home.html). 150 bp paired-end filtered read sequences for *S. purpuratus* generated on the Illumina Genome Analyzer IIx (SRR446979, SRR446980 and SRR446981), for *S. franciscanus* and *A. fragilis* generated on the 454 GS LX (SRR000321–331 and SRR000291–296) were downloaded from the NCBI short read archive (SRA) and converted to FASTQ using fastq-dump from the SRA toolkit (v2.1.10). Sanger sequences were obtained from multiple individuals from various species and used to assess the accuracy of our NGS alignments. For *Ebr1*, *SoxB2* and *CycD* we used data obtained by cloning and sequencing one allele per individual as described in [25, 38]. For the remaining six gene regions, PCR products were sequenced directly using primers listed in (Additional file 1: Table S1).

### *S. purpuratus* Reference genome annotations

We used version 3.1 of the *S. purpuratus* assembly (Baylor College of Medicine Human Genome Sequencing Center Spur v3.1) as the reference genome for our study [40, 41]. An updated Sea Urchin UCSC Genome Browser [48], strPur4, was constructed for the assembly (http://genome-preview.ucsc.edu/cgi-bin/hgGateway?org=S.+purpuratus&db=strPur4; see Additional file 2: Figure S1). A total of 28,965 gene models were obtained from Build 7 of SpBase.org for Spur v3.1 (GLEAN_3_1_Build7). Gene model annotations provided by SpBase.org with the Build 7 genes were used to filter the gene models. 19,546 genes were retained after filtering on these annotations based on the following criteria: we included genes identified as “manually annotated” and having had a “gene model check”, and excluded genes identified with an “un-annotated” or “null” type. A total of 3826 gene models were identified that were not multiples of 3 and excluded. These filters resulted in the identification of 15,850 gene models for further analyses.

### Short read quality control

Data processing was performed using BioPerl (http://bioperl.org), BioPython (http://biopython.org), the Newick Utilities [49], the R statistical package [50], SAMtools [51], BEDTools [52], pysam (https://github.com/pysam-developers/pysam), twobitreader (https://pythonhosted.org/twobitreader/) and the UCSC Genome Browser and source code [53]. Additional tools are explicitly described elsewhere. Job scheduling was done using the Open Grid Scheduler (http://gridscheduler.sourceforge.net/) and parasol (http://users.soe.ucsc.edu/~kent/src/parasol/).

Raw Illumina reads flagged by Casava (Illumina Inc., San Diego, CA) as “filtered” were discarded using the fastq_illumina_filter (http://cancan.cshl.edu/labmembers/gorfon/fastq_illumina_filter). Adapters were identified from a list collected from Omicsoft (https://www.omicsoft.com/downloads/ngs/contamination_list/v1.txt) and trimmed using fastq-mcf from EA-utils v1.1.2–537 (https://code.google.com/p/ea-utils/). The qualities of the libraries pre- and post-filtering were assessed using FastQC (http://www.bioinformatics.bbsrc.ac.uk/projects/fastqc/).

### Short read alignments to the *S. purpuratus* reference genome

Paired-end reads were aligned to the *S. purpuratus* reference genome (Spur v3.1) using Sequence Search and Alignment by Hashing Algorithm (SSAHA2) v.2.5.5 [54]. Although it is an older and slower aligner, we chose SSAHA2 because it provides accurate alignments of short reads to genomes with high levels of heterozygosity due to its flexibility in the degree of mismatches allowed [55–57]. For the paired-end short reads, the assembly hash table was generated using the “-solexa” option. Paired-end alignments with SSAHA2 were performed with an insert length interval of 20–3000 bp, the “-solexa” option and BAM files as output. BAM alignments were merged and sorted using samtools [58]. Short read sequence alignment BAM files are available from the corresponding author upon request.

### Protein-coding alignments

Protein-coding sequences for all gene models were generated using the global nucleotide alignments of each species (including the *S. purpuratus* short reads) to the *S. purpuratus* reference genome. The resulting consensus alignments for all species remained in the reference genome coordinate system (i.e., mapped to the reference). Sharing a single gene model controls for annotation differences between aligned sequences, which may contribute to misalignments and erroneous inference of positive selection [59]. Although a major limitation of reference-based assembly is the introduction of reference bias causing a tendency to underestimate difference between the reference and the aligned genome (e.g., segmental insertions and rearrangements), this can be addressed by limiting our analyses to sites reliably inferred for all species in the alignment at each gene [60]. Post-alignment filtering and masking unreliable sites has been found to be a conservative approach to detecting positive selection [61, 62]. For each gene model, aligned reads for a given taxon were collected for that interval from the BAM alignment file. Pileups were generated using the pysam interface to samtools. Reads were discarded if they were not properly paired or had mapping quality scores <25. Insertions not present in the reference genome were ignored and deletions were treated as missing data. Reference coordinates of indels and missing data from any species were also recorded. For duplicate reads, we retained those with the highest mapping quality. Variable nucleotide positions were identified and called as ambiguous (using IUPAC codes) for sites with a minimum observed allele frequency of 0.125 and a minimum coverage of 8 filtered reads. For all gene models in each species we tabulated total base calls, numbers of duplicate reads, numbers and frequencies of heterozygous sites, and counts of sites containing >2 bases. Per-site coverage depth and mean coverage was also determined. Gaps and ambiguities were retained in the alignments but were filtered prior to tests for positive selection (see below). We assign missing data (i.e. “N”) for any gene model reference position in a given species sequence when we detect either (i) a no call based on our base calling algorithm due to a missing or unsupported allele call, or (ii) an indel (insertion or deletion) reported by the BAM alignment file.

Protein-coding alignments containing paralogs (i.e., reads from two or more loci mapping to the same *S. purpuratus* reference gene) or spurious artifacts (e.g., alignment errors) were identified and removed from the data by applying three filters. First, high quality nucleotide positions containing >2 bases were identified across all sites in all species. Second, alignments were flagged in a species that contained a large increase in coverage (> 2 S.D.) compared to the genome-wide mean exon coverage for that species. Finally, we checked for skews in the frequencies of mutations away from that expected for a true heterozygous site (*p* = 0.50) to that expected for heterozygous mutations present in paralogs (i.e., *p* = 0.25 or 0.75 for the case of two paralogs). For each alignment in all species we tabulated the counts of heterozygous mutations falling into three allele frequency bins (low 0.125–0.375, intermediate 0.375–0.625, and high 0.625–0.875) and recorded when a greater number of mutations fell in either the low or high bins compared to the intermediate bin. The robustness of these filters were assessed by the visual inspection of 238 alignments containing putative paralogs and 79 alignments deemed free of paralogs. Protein-coding consensus sequence alignments are available in Additional file 3.

### Tests for positive selection

Tests for positive selection were performed using the codeml program of PAML v.4.5 [18]. For all single-copy alignments, we ran PAML sites models M0, M7 and M8. For all runs, we input maximum likelihood gene trees obtained by PhyML v.20110919 [63] using a GTR model [64], the estimated rate and probability of each class from the data (“free_rates”), optimized tree topologies, branch lengths and rate parameters, and the best tree topology of NNI and SPR search operations. Equilibrium codon frequencies were calculated from the average nucleotide frequencies at the three codon positions (F3X4). Kappa and *d*_N_/*d*_S_ ratios were estimated from the data using initial values of 1.6 and 0.4, respectively.

Since we were interested in testing for fixed differences between species driven by positive Darwinian selection, codons with ambiguous (i.e., heterozygote) sites were removed. To minimize the false inference of positive selection caused by misalignment or annotation errors (e.g., [59, 65]), all alignments with fewer than 100 codons for all nine species were discarded and those remaining were cleaned by PAML to remove gaps and missing data. The loss of power caused by this filtering is more than compensated by the increased accuracy of correctly identifying positively selected genes [62]. To retain genes that failed PAML testing due to the presence of premature stop codons, we trimmed 10 codons from the end of the *S. purpuratus* reference gene and re-ran the three codeml models. Candidate positive selected codons (PSCs) were identified by the Bayes Empirical Bayes approach of [66] and translated to the genomic coordinates for analysis and presentation. Wiggle tracks for PSCs were created and reported as the –log_10_ (*P-value*).

*P*-values for the M8 vs. M7 likelihood ratio tests were generated from an empirical distribution of scores following the approach of [7]. We randomly selected 650 alignments and collected the estimated *d*_N_/*d*_S_ ratios, kappas, codon frequencies, tree lengths and tree topologies from the model M7 output. Based on these parameters, the evolverNSsites program of the PAML package was used to generate 11,700 simulated alignments (18 replicates for the 650 alignments) for each null model. Models M7 and M8 were then run on each simulated alignment to produce an empirical null distribution of likelihood ratio test (LRT) scores. The empirical null distribution was used to generate *P*-values for the M7 vs. M8 test for positive selection using the “ecdf” function in R.

To correct for multiple hypothesis testing, we controlled the false discovery rate (FDR) by calculating q-values using the “fdrtool” package in R [67]. The q-values were calculated using the default empirical null model in “fdrtool” [68] using the *P*-values obtained from the empirical null distribution of LRT scores. Candidate positive selection genes (PSGs) were identified at a FDR of 5%.

Enrichment tests were conducted on the GO terms if Build7 collected from SpBase.org [41] and on the 20 major functional classes (L1) and 56 subclasses (L2) identified by [42]. Tests were not conducted on the additional subclasses (L3) because of their considerable overlap with the L2 groupings. *P*-values for the enrichment of genes experiencing positive selection for different GO terms and functional groupings of [42] were calculated using the hypergeometric (HG). To minimize the possibility of inflated scores for small gene sets, GO terms having <20 genes or having only a single PSG were discarded. We assessed the FDR using q-values based on the HG-derived *P*-values. Functional groupings with only a single PSG were excluded. The one-tailed *P*-values for the enrichment of genes experiencing positive selection in a priori candidate categories were determined by Fisher’s exact tests for independence using “fisher.test” in R.

We also implemented branch-sites tests of positive selection to identify lineage-specific episodes of adaptive protein evolution [66, 69]. To be conservative, one test was performed for each terminal branch leading to the nine extant taxa using the inferred gene tree. Because the *P*-value distribution for branch-sites tests is not uniform, the test statistic for the null-alternative model comparisons was a 1:1 mixture of point mass 0 and χ_1_^2^ [20, 70]. We corrected for multiple tests using Bonferonni correction of the family-wise error rate for each branch tested for that gene model [71]. Enrichment tests for GO terms of PSGs identified along terminal branches were performed for GO terms having at least a single PSG and we applied a FDR of 10%.

### Consensus sequence alignment validation

Recently, several studies have observed that genome-wide tests for positive Darwinian selection suffer from excessive false positives due to annotation and alignment errors [59, 61, 65]. In the Drosophila 12 genomes data, [59] observed that many PSCs occurred within 15 amino acids of an exon border or near regions containing insertions and deletions of amino acids. Due to the small size of exons in *S. purpuratus* (median = 48 amino acids), we tested for significant enrichment of PSCs within 3, 5 and 7 amino acids of an exon border and adjacent to regions (both upstream and downstream) containing an indel or missing data (presumably due to deletions or alignment errors). We also performed visual inspections of the top 64 PSGs to identify the locations of PSCs using the UCSC Sea Urchin Genome Browser (http://genome-preview.ucsc.edu/cgi-bin/hgTracks?db=strPur4). Finally, alignments from Sanger sequences from ten gene regions were collected and compared with those generated from the Illumina data.

## Results

### Sequencing, alignments, and paralog filtering

**Fig. 1:**
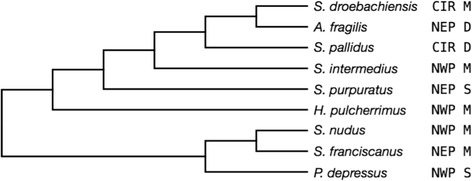
Phylogeny of nine species of strongylocentrotid urchins examined in the present study (reproduced from [44]). The species tree was generated from four-fold degenerate sites from 2301 concatenated genes not exhibiting positive selection. Bayesian, maximum-likelihood, and maximum parsimony trees produced identical topologies. Next to each species is information on their distributions (CIR = Circumpolar, NWP = North West Pacific, NEP = North East Pacific) and adult depth ranges [S = Shallow (0–50 m), M = Medium (0–200 m), D = Deep (0–1600 m)]

The results of the short read paired-end sequencing of the nine sea urchin genomes are summarized in Table 1. The numbers of post-filtered reads varied from 146.3 million (*S. pallidus*) to 373.3 million (*S. intermedius*). For all species, the percentage of bases in the *S. purpuratus* reference genome covered with at least one read scaled with phylogenetic position relative to the reference (see Fig. 1). Mean coverage across the complete genome typically ranged from 30 – 40X and increased to >50X for single-copy protein coding genes with the notable exception of *S. pallidus*. Coverage for *S. pallidus* was ~1/3 lower due to its genome being sequenced during the early developmental phase of the automated library preparation protocols.

**Table 1:** Summary of genomic DNA sequencing and alignment coverage

						Mean coverage	
Species	Illumina platform	Read length	No. reads post-filter	No. properly paired mates	% Bases covered^a^	Complete genome	Single copy orthologs^b^
*S. droebachiensis*	HiSeq 2000	100 bp PE	292,505,508	207,144,870	77.4	35.1X	57.6X
*A. fragilis*	HiSeq 2000	100 bp PE	373,338,930	269,556,782	80.7	45.0X	58.9X
*S. pallidus*	HiSeq 2000	100 bp PE	146,289,354	94,967,354	77.7	16.2X	16.2X
*S. intermedius*	HiSeq 2000	100 bp PE	333,848,336	241,604,806	76.9	39.7X	57.9X
*H. pulcherrimus*	HiSeq 2000	100 bp PE	327,231,340	216,769,754	71.2	39.2X	63.5X
*S. nudus*	HiSeq 2000	100 bp PE	348,104,257	223,613,387	64.1	41.9X	67.0X
*S. franciscanus*	HiSeq 200	100 bp PE	323,937,718	240,552,762	53.9	39.2X	52.3X
*P. depressus*	HiSeq 2000	100 bp PE	308,218,933	189,969,224	56.8	37.1X	91.9X
*S. purpuratus*	GA IIX	150 bp PE	239,280,430	164,442,076	96.8	44.8X	n.d.c

^a^Percentage of bases in the *S. purpuratus* reference genome covered with at least one read

^b^Mean coverage across the protein-coding sequences of 6520 single-copy orthologs

^c^not determined

Based on the Spur v3.1 release of the *S. purpuratus* reference genome (SpBase.org builds 6 and 7) we obtained initial alignments for 15,850 gene models. Alignments containing stop codons, missing data from one or more species, or having fewer than 100 codons [7] were removed from the data (resulting in 9015 genes). Our decision to apply a 100-codon cutoff served two important purposes. First, it allowed us to compare all genes tested at a minimum size known to provide adequate statistical power [19]. If this cutoff was not applied, tests would have performed on alignments with variable numbers of codons below 100 that would have resulted in an unknown number of non-significant likelihood ratio tests (LRTs) caused by low statistical power. The 100-codon cutoff ensured that this problem was avoided. Second, *P*-values for the tests for positive selection were based on an empirical distribution of statistics (see below) [7]. Parameters for these simulations included tree topologies, tree lengths, codon frequencies, kappas and dN/dS ratios. Applying a 100-codon cutoff ensured that the parameters used for these simulations minimized any inaccuracies caused by small alignments.

We applied three criteria to identify and eliminate alignments containing artifacts: (i) the presence of nucleotide sites containing >2 mutations, (ii) a dramatic increase in coverage (>2 S.D.), and (iii) a skew in heterozygote allele frequencies away from 0.50 (see Methods for details). Visual inspection of alignments confirmed that the vast majority of alignment artifacts were caused by paralogous sequences mapping to the same reference gene. We observed >2 alleles at 231 of 238 alignments flagged as containing putative paralogs and found no discrepancies at 79 alignments deemed free of paralogs. Our three metrics showed a high degree of overlap when paralogs were present in an alignment (see Additional file 1: Table S2) and were used to conservatively eliminate 2495 genes from further analyses (1483 containing putative outparalogs and 1012 with putative inparalogs). These filters resulted in final data set of 6520 single-copy orthologs free of known alignment artifacts that were tested for positive selection.

### Positive selection

**Fig. 2:**
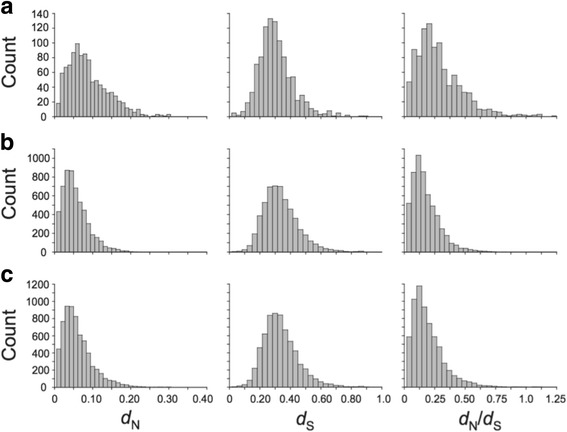
Distributions of rates of nonsynonymous (*d*_N_) and synonymous (*d*_S_) substitutions and *d*_N_/*d*_S_ ratios for the **a** positive selected genes (PSGs; *n* = 1008), **b** genes not showing positive selection (non-PSGs; *n* = 5512), and **c** all genes (*n* = 6520)

Using maximum-likelihood models of codon substitution implemented by PAML [18], we detected strong signals of positive selection across the nine sea urchin genomes. Fitting models M7 and M8 and applying a false discovery rate (FDR) of 5%, we identified 1008 (15.5%) candidate positively selected genes (PSGs) (Table 2 and Additional file 4). Increasing the FDR to 10% resulted in a modest increase in the number of PSGs to 1529 (23.5%). The signal of positive selection in our data was clearly attributable to significant elevations of both *d*_N_ and *d*_N_/*d*_S_ ratios in the PSGs by ~70% (Mann-Whitney U test *P* < 2 × 10^−16^) (Fig. 2). By contrast, mean *d*_S_ did not differ significantly between genes experiencing positive selection (0.333) and those showing no evidence for positive selection (0.340) (Mann-Whitney U test *P* = 0.0581). The largest *d*_N_ and *d*_N_/*d*_S_ ratios were observed in the most strongly selected PSGs but *d*_S_ showed no similar trend (Additional file 2: Figure S2). The increased rate of nonsynonymous substitution at the PSGs also resulted in a 33.5% reduction in the numbers of heterozygous mutations relative to the non-PSGs (Additional file 1: Table S3). This reduction in heterozygosity was not an artifact of filtering because (i) the majority of codons were removed from alignments because of missing data (i.e., not heterozygote sites), and (ii) a higher proportion of codons containing ambiguous sites were filtered from the PSGs (14.8%) than the non-PSGs (10.2%) (Additional file 1: Table S4). The mean number of codons analyzed was also significantly higher in the PSGs (Mann-Whitney U test *P* < 2 × 10^−16^) (Table 2; Additional file 2: Figure S3) suggesting that our ability to detect positive selection was constrained by protein size (or by the extent of missing data) as documented by [19]. Interestingly, the non-PSGs exhibited a higher proportion of codons exhibiting positive selection (5.44%) compared to the PSGs (3.99%) but this difference was not significant (Mann-Whitney U test *P* = 0.096) (Table 2).

**Table 2:** Comparison of candidate positively selected genes (PSGs) and those not exhibiting positive selection (non-PSGs)

Category	No. of genes	No. of base pairs	Mean No. of codons	Mean p1 M8^a^	Mean LRT Score^b^	Mean *d*N	Mean *d*S	Mean *d*N*/d*S^C^
PSGs	1008	2,188,170	502.4	0.0399	13.67	0.0844	0.333	0.253
Non-PSGs	5512	8,487,210	341.8	0.0544	1.35	0.0503	0.340	0.148
TOTAL	6520	10,675,380	366.6	0.0521	3.26	0.0556	0.339	0.164

^a^Mean percentage of codons with *d*_N_ */d*_S_ ratios >1 identified by PAML model M8

^b^Mean Likelihood Ratio Test Score comparing PAML models M7 and M8

^c^Calculated following Wolf et al. (2009)

In Drosophila, [59] showed that alignment errors in regions containing insertions/deletions and at exon boundaries commonly generated false signals of positive selection. In the present study, 956 of the 1008 PSGs had at least one positively selected codon (PSC) with a posterior probability >0.95. In these 956 genes, we tested for significant enrichment of PSCs in 3, 5, or 7 amino acid windows adjacent to exon boundaries and next to regions containing indels or missing data (potentially caused by alignment errors). We observed the opposite pattern to that reported in Drosophila; amino acids close to exon boundaries and indel/missing data regions exhibited significant under-enrichments of PSCs for all window sizes tested (Additional file 1: Table S5 and S6). We manually inspected 64 alignments containing PSCs and confirmed that positive selection typically occurred in regions lacking gaps (11 examples are shown in Additional file 2: Figure S4 – S14). To further check the accuracy of our alignments, we collected and aligned Sanger sequences from 10 gene regions and compared these alignments with the short read data. No differences were observed between the Sanger and Illumina alignments for genes containing small gaps, having many mismatches to the reference, or possessing moderate to high numbers of heterozygous sites (Additional file 2: Figure S15 – S23). However, regions of proteins containing highly divergent repeat motifs were masked in short read alignments but were present in the Sanger data (Additional file 2: Figure S24).

Codon bias and non-random patterns of synonymous codon usage have been recently described in *S. purpuratus* [46] that, if present in other species, has the potential to bias *d*_S_ and *d*_N_ */d*_S_ ratios. However, measures of codon bias and GC content across the protein-coding gene regions were indistinguishable between the PSGs and those not exhibiting positive selection (Additional file 1: Table S7, and Additional file 2: Figure S3). Our data set also contained 542 genes lacking introns and 710 hypothetical proteins. The percentage of genes lacking introns did not differ between the PSGs (9.13%) and those not experiencing positive selection (8.16%) (Fisher’s exact test, *P* = 0.321). However, similar to that described in *Drosophila* [22] hypothetical proteins were significantly overrepresented in the PSGs (15.7%) compared to the non-PSGs (10.0%) (Fisher’s exact test, *P* < 0.0001). Positively selected hypothetical proteins were significantly larger, had significantly higher *d*_N_ and *d*_N_ */d*_S_ ratios and significantly lower *d*_S_ than hypothetical proteins not showing positive selection (Mann-Whitney U tests *P* < 0.0001, *P* < 0.0001, *P* < 0.0001, and *P* = 0.0274, respectively) (Additional File 1: Table S8). However, hypothetical proteins showing positive selection exhibited indistinguishable levels of codon bias, kappa and GC content from those not showing positive selection the non-PSGs (Additional file 1: Table S9).

We detected significant enrichment of positively selected genes for 9 GO terms (Table 3). Membrane proteins associated with hemophilic cell adhesion, transport activity, receptor activity, or binding activity were predominant members of this group. Enrichment tests for positively selected genes in the functional categories defined by [42] also identified adhesion proteins as the most strongly selected group (Table 4 and Additional file 1: Table S10). Significant enrichment was observed for the “Metabolism_Inorganic Ion” subcategory attributable to positive selection at 8 potassium channels, 7 solute carriers and 6 co-tranporters. Although positive selection was clearly evident at these functional categories, the majority of GO terms exhibited mean *d*_N_ */d*_S_ ratios less than 0.20 confirming a dominant role of purifying selection (Additional file 2: Figure S25, S26, and S27).

**Table 3:** Gene ontogeny (GO) categories significantly enriched in genes experiencing positive selection

Category	Description	No. of genes			Fold enrichment^c^	*P*-value^d^
		Tested	PSG^a^	E[PSG]^b^		
Molecular Function						
GO:0004252	Serine-type endopeptidase activity	36	15	5.6	2.7	0.0003
GO:0004930	G-protein coupled receptor activity	45	15	7.0	2.2	0.0020
GO:0005328	Neurotransmitter sodium symporter activity	17	9	2.6	3.4	0.0001
GO:0005509	Calcium ion binding	140	42	21.6	1.9	<0.0001
GO:0008484	Sulfuric ester hydrolase activity	23	10	3.6	2.8	0.0013
Cellular Component						
GO:0016020	Membrane	546	120	84.4	1.4	0.0001
Biological Process						
GO:0006508	Proteolysis	147	36	22.7	1.6	0.0027
GO:0006836	Neurotransmitter transport	17	9	2.6	3.4	0.0002
GO:0007156	Homophilic cell adhesion	14	8	2.2	3.7	0.0003

^a^Positively Selected Gene

^b^Expected number of PSGs

^c^Fold enrichment of observed PSGs to E[PSGs]

^d^Empirical *P*-values were determined by the hypergeometric with 10,000 re-samplings from a total of 6520 genes tested and 1008 identified as PSGs. All remain significant at a False Discovery Rate of 10%

**Table 4:** Significant enrichment tests for genes experiencing positive selection using the Tu et al. (2012) custom sea urchin gene ontology (GO) level 2 (L2) functional categories

		Number of genes				
Category	Subcategory	Tested	PSG^a^	E[PSG]^b^	Fold enrichment^c^	*P*-value^d^
Adhesion	Adhesion_ECMCollagen	15	10	2.3	4.3	<0.0001
	Adhesion_ECMFibropellin	5	3	0.8	3.9	0.0632
	Adhesion_ECMNeural	13	5	2.0	2.5	0.0690
	Adhesion_ECMReceptorCadherin	14	8	2.2	3.7	0.0013
	Adhesion_ECMReceptorIgFN3	21	9	3.2	2.8	0.0056
Biomineralization	Biomineralization_Collagen	6	4	0.9	4.3	0.0168
Defensome	Defensome_TransporterABC	19	6	2.9	2.0	0.0928
Immunity	Immunity_ReceptorScavenger	21	7	3.2	2.2	0.0566
Metabolism	Metabolism_InorganicIon	107	27	16.5	1.6	0.0158
Metalloprotease	Metalloprotease	84	20	13.0	1.5	0.0599
Signaling	Signaling_Notch	10	4	1.5	2.6	0.0947

^a^Positively Selected Gene

^b^Expected number of PSGs

^c^Fold enrichment of observed PSGs to E[PSGs]

^d^ Empirical *P*-values were determined by the hypergeometric with 10,000 re-samplings from a total of 6520 genes tested and 1008 identified as PSGs

The top 15 candidate positively selected genes are listed in Table 5. All loci exhibited elevated *d*_N_ */d*_S_ ratios and, although highly variable in size, had a greater mean length (707.9 codons) than the average single-copy gene (366.6 codons). The majority of proteins (10) were either extracellular matrix proteins or integral membrane proteins, including the egg receptor for bindin (*EBR1*). Genes functioning in the innate immune system were absent from the top-ranked PSGs. The strongest signal of positive selection was observed at type IV collagen (*3Apcol*; 27 exons), a constituent of the basement membrane. A second gene model for type IV collagen (*3Acolf*; 15 exons) appeared in the list that overlapped with the first 5 exons and two positively selected codons of *3Apcol*. Overlapping gene models showing positive selection were rare in our data, however, occurring in only 9 of the 1008 candidate PSGs. It is interesting to note that fibropellins, fibrosurfins, usherins, and EMI/Egf proteins are all known to interact with type IV collagen [72, 73].

**Table 5:** Summary of the top 15 ranked positively selected genes (PSGs)

Rank	SPU gene	Name	Protein	Location^a^	No of codons	*d*N	*d*S	*d*N*/d*S	LRT Score^b^	No. of positively selected codons^c^
1	SPU_003768	Sp-3Apcol	Type IV collagen	ECM	1183	0.159	0.345	0.459	233.04	77
2	SPU_010829	Sp-Ef2	Elongation factor 2	RIB	800	0.153	0.473	0.322	102.62	20
3	SPU_008159	Sp-Kcnk13	Potassium channel	MEM	405	0.079	0.209	0.378	91.68	11
4	SPU_018532	Sp-EgfibL	Fibropellin 1b–like	ECM	439	0.268	0.307	0.873	79.67	38
5	SPU_006534	Sp-Ebr1_5	Egg bindin receptor 1–5	MEM	691	0.174	0.368	0.473	66.79	12
6	SPU_008462	Sp-Hypp_548	Usherin-like	ECM	684	0.212	0.298	0.710	61.85	21
7	SPU_006645	Sp-Gdi1_1	GDP dissociation inhibitor 1	CYT	184	0.254	0.278	0.914	61.38	12
8	SPU_000526	Sp-Ebr1	Egg bindin receptor 1	MEM	1428	0.185	0.518	0.357	59.84	9
9	SPU_009154	Sp-Hypp_42	Unknown protein	UNK	1017	0.217	0.346	0.629	58.28	19
10	SPU_003671	Sp-Lrp12	Low density lipoprotein	ECF	761	0.143	0.453	0.315	55.58	13
11	SPU_018517	Sp-Hypp_915	Fibrosurfin-like	ECM	424	0.282	0.346	0.814	54.02	15
12	SPU_002551	Sp-Egfiii	Fibropellin c	ECM	357	0.194	0.415	0.468	53.88	6
13	SPU_016836	Sp-EMI/Egf	MEGF10-like	MEM	1709	0.110	0.271	0.408	53.41	7
14	SPU_005187	Sp-3Acolf	Type IV collagen	ECM	384	0.116	0.289	0.400	51.59	5
15	SPU_003825	Sp-14-3-3e	14–3-3 epsilon	CYT	152	0.090	0.255	0.354	51.20	4

^a^Cellular location of protein. *ECM* extracellular matrix, *ECF* extracellular fluid, *MEM* membrane or intrinsic to membrane, *CYT* cytosolic, *RIB* ribosome, *UNK* unknown

^b^Mean Likelihood Ratio Test Score comparing PAML models M7 and M8

^c^Number of codons with Bayes Emperical Bayes posterior probabilities >0.95

### Positive selection along terminal branches

Branch-sites tests were performed to detect the presence of positive selection along the nine terminal branches of the strongylocentrotid phylogeny (Table 6). Significant results were observed at only 824 (1.67%) out of 49,460 individual tests. The greatest numbers of positively selected loci were observed on the *S. pallidus*, *S nudus* and *P. depressus* terminal branches, which together accounted for 46.2% of all significant tests. Only one third of genes (277 of 824) exhibiting significant branch-sites tests overlapped with those showing significant positive selection summarized in Table 2, and 141 (17.1%) significant tests occurred in the bottom third of genes ranked by the strength of positive selection (i.e., the M8 vs. M7 LRT score) that were characterized by a strong overall signal of purifying selection. However, 12 of the 15 of the top positively selected genes listed in Table 5 also showed significant branch-sites tests (missing were *Gdi1_1*, *EMI/Egf*, and *14–3-3e*).

**Table 6:** Summary of the branch-sites test results

Species	No. of tests	Minimum LRT score	No. of sig. test	No. overlapping with sites tests^a^	No. of GO categories tested	No. of significantly enriched GO categories
*S. droebachiensis*	5343	5.629	85	23	113	18
*A. fragilis*	5283	5.759	64	18	90	29
*S. pallidus*	5300	5.788	143	54	166	49
*S. intermedius*	5340	6.038	79	28	89	22
*S. purpuratus*	5383	6.253	57	17	47	16
*H. pulcherrimus*	5762	5.891	87	26	89	26
*S. nudus*	5652	5.525	118	42	139	25
*S. franciscanus*	5472	6.296	71	25	106	32
*P. depressus*	5925	6.033	120	44	121	26

^a^Number of PSGs also showing significant branch-sites tests

Along the terminal branches of the nine species, we tested 960 GO terms for enrichment (Table 6). Applying a FDR of 10%, we observed a total of 243 significantly enriched GO terms (summarized in Additional file 1: Table S11). The most consistent signals of selection were observed at membrane or integral membrane proteins and those that functioned in protein binding, ATP binding, or zinc ion binding. The most divergent lineage was *S. pallidus* that had 32 unique enriched GO terms, which was more than double that of the other eight species combined. The four western Pacific species tended to have more significant branch-sites tests (mean = 97.3) than the three eastern Pacific species (mean = 69.0) but the adult depth distributions appeared unrelated to numbers of significant tests.

## Discussion

Comparative genomics studies testing for positive Darwinian selection among groups of closely related species are still few in number and taxonomically limited (e.g., [8, 22, 74, 75]). The present study assessed the magnitude and the targets of positive selection in nine north temperate sea urchins using whole-genome sequencing and the well-annotated genome of the purple sea urchin, *S. purpuratus*, as a reference. Applying a conservative FDR of 5%, we observed that 15.5% of 6520 single-copy orthologs exhibited significant positive selection, confirming a strong signal of adaptive diversification. Branch-sites tests identified 824 candidate genes experiencing positive selection along the nine terminal branches of the phylogeny (1.67% of the tests performed). The positive selection detected across the sea urchin genomes was clearly attributable to an increased rate of nonsynonymous substitution (*d*_N_). Mean *d*_N_ was 68% higher and mean heterozygosity 33% lower in the candidate positively selected genes (PSGs) compared to genes not showing positive selection. This reduction in heterozygosity is not an artifact of filtering (Additional file 1: Table S2) and opposite to that expected by a relaxation of selective constraint, but is consistent with the predicted effects of selective sweeps reducing linked variation in the vicinity of the selected sites. Rates of synonymous substitution (*d*_*S*_), GC content, and codon bias were similar among all genes tested thus minimizing any spurious inflation of *d*_N_ */d*_S_ ratios.

The sea urchin species examined in our study possess high levels of genetic variation, large effective population sizes, extensive geographic ranges, and minimal population structure [37–39]. Theory predicts that these species attributes should result in low *d*_N_ */d*_S_ ratios due to the increased effectiveness of both purifying selection and directional selection in large populations (Ohta 1992). Previous studies on mammals [8] and *Drosophila* [22] have generally supported the prediction that *d*_N_ */d*_S_ ratios vary inversely with effective population size [23, 76]. Our mean *d*_N_ */d*_S_ ratio (0.164) is lower than observed in humans (0.249) or chimpanzees (0.245) but higher than mouse (0.127) or rat (0.121) [8]. Surprisingly, our median *d*_N_ */d*_S_ ratio (0.144) is considerably larger than reported in *Drosophila* (median = 0.064; [77]). It is possible that weak selection acting on synonymous codon usage (cf. [46]) might have reduced *d*_S_ and hence inflated *d*_N_/*d*_S_. However, many non-PSGs had codons exhibiting weak positive selection but failed to generate significant likelihood ratio tests and spurious inflation of *d*_N_ */d*_S_ ratios were minimized (i.e., rates of synonymous substitution (*d*_*S*_), GC content, and codon bias were similar among all genes tested). Thus, the elevated median *d*_N_ */d*_S_ ratio is more likely caused by an increased substitution rate of adaptive nonsynonymous mutations. Our results suggest that selective sweeps may be common in natural populations of sea urchins, a prediction that could be tested by examining the patterns of neutral diversity and linkage disequilibrium around positively selected amino acid positions identified in our study (e.g., [16, 17]).

Although we observed strong positive selection across the nine sea urchin genomes, there are several reasons why our results are likely conservative. First, we were unable to analyze several large multi-gene families expected to show positive selection because of the prevalence of alignments containing paralogs. Notable here are genes functioning in the sea urchin innate immune system, particularly Toll-like receptors (TLRs), NACHT domain and leucine-rich repeat proteins (NLRs) and scavenger receptor cysteine-rich proteins (SRCRs), which exhibit positive selection in *Drosophila* [77, 78]. The TLR, NLR and SRCR gene families each have ~200 loci in the Spur v.3.1 genome assembly [79]. Our initial filters included 217 innate immunity genes but only 29 were tested for positive selection after the removal of alignments containing paralogs. Visual inspection of the alignments for the 188 dropped gene models on the updated UCSC Sea Urchin Genome Browser (http://genome-preview.ucsc.edu/cgi-bin/hgTracks?db=strPur4) found that ~70% had paralogs in all species confirming that these genes are experiencing extensive gains and losses similar to that described in *Drosophila*. Second, our tests for positive selection excluded all codons containing any heterozygous mutations from the data (representing 1.72 million high-quality SNPs). To examine the impact of this filtering on the detection of positive selection, we applied parsimony criteria to call heterozygous mutations and retain codons in the top 25 PSGs and in 25 genes just below the 5% FDR cut-off (Additional file 1: Table S12 and S13). As expected, the signals of positive selection increased in both groups when more codons were tested. Third, we observed that larger proteins exhibited much stronger positive selection than smaller proteins suggesting that low statistical power had constrained our ability to detect selection on the latter (see [19, 80]). Power would be further impacted by the loss of roughly a third of all codons by filtering heterozygous positions from the data.

Several recent studies have found that errors in sequencing, annotation and alignment can generate significant spurious signals of positive selection [59, 61, 65, 81]. There are several reasons why the high rates of false positives reported in the mammalian and *Drosophila* genomes have been minimized in our study. First, we used a reference-based alignment method that excluded all codons not present in the *S. purpuratus* reference genome (caused by insertions) and removed all missing codons (caused by deletions) prior to testing for positive selection. Masking to the *S. purpuratus* reference genome should have largely avoided alignment errors involving insertions or deletions of amino acids. Second, we performed strong quality control filters on the well-characterized protein-coding genes of *S. purpuratus* to minimize annotation errors (i.e., incorrect or inconsistent start/stop codons and exon/intron boundaries). Third, we applied strict alignment and base quality metrics and high coverage cut-offs to exclude potential sequencing error. Fourth, all codons containing heterozygous mutations were excluded from the analyses, which eliminated spurious elevation of *d*_N_ that would have resulted from incorrectly calling heterozygous mutations as fixed. Our estimates of *d*_S_ were also well below saturation (only 8.4% of loci had *d*_S_ values >0.50) and unrelated to the detection of positive selection. Fifth, we observed no significant enrichment of PSCs in regions found to contribute to false-positives in other studies (i.e., intron/exon boundaries and gene regions containing adjacent to missing data and/or insertions/deletions). Finally, Sanger sequencing and the manual inspection of many genes confirmed that positive selection invariably occurred in regions free of alignment gaps (Additional file 2: Figure S4 – S24).

Our study revealed a number of unusual targets of positive selection. The strongest signal of positive selection was observed at type IV collagen (*3Apcol*), a basement membrane protein at which 77 amino acids exhibited significantly elevated *d*_N_ */d*_S_ ratios. An additional 11 codons had posterior probabilities between 0.90 and 0.95 and a further 90 unique nonsynonymous mutations had fixed across the nine sea urchin species. To our knowledge, positive selection has not been previously described at any collagen gene. Although this might indicate a relaxation of selective constraint on *3Apcol*, our results clearly show that the protein is experiencing both strong purifying and potent diversifying selection. Type IV collagen is a right-handed triple helix containing a repeating unit of three amino acids (Gly-X-Y) where ‘X’ and ‘Y’ are commonly proline or hydroxyproline [82, 83]. Our *3Apcol* alignment contained 387 collagen repeats and the amino acid sequences of 241 triplets were identical. Remarkably, first-position glycines in all 387 Gly-X-Y repeats were completely conserved in all nine species. This confirms the action of strong purifying selection on first-position glycines and diversifying selection on a subset of second and third repeat positions. Five positively-selected residues occurred in noncollagenous domains (i.e., outside Gly-X-Y repeats) and the remainder were equally divided between second and third repeat positions (37 and 35, respectively) distributed over most of the protein.

Positive selection was also detected at several genes implicated in sexual conflict or sexual selection. Positive selection has been previously documented at *bindin* and one *EBR1*exon in four strongylocentrotid sea urchins [25]. Concordant with these previous findings, we found both the egg receptor for bindin (*EBR1*) and the sperm protein gene *bindin* exhibited positive selection, although the latter appeared well down our list (ranked 913). Another putative egg receptor protein gene (*EBR1_5*) ranked ahead of *EBR1* in our top candidate PSGs. The EBR1_5 protein (1243 amino acids) contained a transmembrane domain, a SEA domain and seven hyalin repeat (HYR) domains. Positive selection was restricted to three HYR domains, notably the 3rd repeat where seven positively selected codons clustered in a stretch of 76 amino acids. The detection of selection at both *EBR1* and *bindin* was likely underestimated because both gene models were incomplete (resulting in a loss of data and hence statistical power) and sections of both proteins containing functionally important repeat units could not be robustly aligned. A more detailed treatment of the coevolution between *EBR1* and *bindin* will be addressed in a separate paper (Kasimatis, Kober, and Pogson, in preparation). Overall, the number of loci involved in sexual selection/conflict appear less numerous than potential pathogen targets and enrichment tests for sperm or egg genes were not significant.

Many of our candidate positive selection genes are extracellular matrix (ECM) proteins, cell-surface receptors, cell adhesion molecules, cytoskeletal elements or proteins that function in signal-transduction or vesicle trafficking pathways (Additional file 1: Table S11). It is difficult to see any obvious connection between this diverse set of proteins and environmental factors such as temperature, depth or diet. However, many of our candidate PSGs have been linked to pathogens in other organismal groups. For example, type IV collagen and other ECM proteins have long been recognized as targets of pathogen binding [84, 85] or proteolytic degradation [86] that facilitate host cell invasion (see recent review by [87]. A wide array of bacterial and viral pathogens are also known to exploit or mimic cell receptors [88], cell adhesion molecules [89], cell cycle and signaling pathways [90], and cytoskeletal proteins like dyneins [91]. Aquatic environments harbor a wide array of toxins (see [92]) and several of our top candidate PSGs are susceptible to these agents. For example, *EF2* (our second-ranked PSG) is the target of ADP-ribosylating diphtheria toxin in humans [93] and a similar acting toxin has evolved independently in the marine pathogen, *Vibrio cholera* [94]. Voltage-gated ion channels (such as our third-ranked PSG, *Kcnk13*) are also commonly inactivated by toxins. Positive selection at *Kcnk13* in sea urchins is restricted to a 43 amino acid segment adjacent to the extracellular entrance to the K^+^channel pore, which is the same region targeted by scorpion venom [95]. Antagonistic coevolution between marine pathogens and their cellular targets could account for much of the positive selection observed in our study.

The importance of pathogens as major drivers of adaptive evolution was originally recognized by Haldane [96]. Recent studies on humans have implicated pathogens as being one of the most important agents of adaptive diversification [14, 97]. Our study suggests that pathogens could also be major selective agents in the marine environment. It is interesting that the strongest signals of positive selection were observed at the putative targets of pathogens, not the genes directly involved in host defense. Although positive selection was detected at some innate immunity genes, these exhibited weaker signatures of selection than many proteins implicated as pathogen targets. This requires further study because we were unable to test the majority of innate immunity genes for positive selection because of the widespread presence of paralogs. The unusual targets of positive selection in sea urchins might reflect a higher pathogen pressure in the marine environment, where organisms are bathed in a medium containing high densities of viruses and other pathogens [98, 99]. Strongylocentrotid sea urchins are prone to disease outbreaks [35, 100], but the ensemble of pathogens they experience remains largely uncharacterized. At the same time, the adaptive diversification of structural proteins might reflect the increased efficacy of selection in sea urchins because of their large effective population sizes. Comparative genomics studies on other marine groups are needed to provide insights into the role of pathogens as selective agents and the importance of population size on the prevalence of positive selection.

Our tests for selection were based on *d*_N_ */d*_S_ ratios that use historical patterns of substitutions across the entire gene tree (sites tests) or more recent adaptation along terminal branches (branch-sites tests). An alternative test for selection is to use genome-wide SNP data to identify F_ST_-outliers among natural populations of a species caused by recent diversifying selection [101, 102]. The relationship between genes experiencing long-term positive selection and those undergoing adaptive differentiation among contemporary populations is not well understood. However, several recent studies in *S. purpuratus* have used genome scans to identify SNPs with significant population differentiation [103–105], thus allowing a comparison between historical and contemporary patterns of selection. Comparing two populations of *S. purpuratus* from the NE Pacific, [104] identified three strong F_ST_-outliers out of 9112 SNPs falling in coding or upstream gene regions. Twenty-six of their top 100 F_ST_-outliers overlapped with the genes analyzed in our study. Six genes were candidate PSGs in our study but none had experienced recent selection in the *S. purpuratus* lineage. In a follow-up study, [105] detected a significant enrichment of F_ST_-outliers in non-coding upstream regions of E3 ligase genes that were correlated SNPs in coding regions. Our tests for positive selection included 33 E3 ligase genes but only one (SPU_027523) exhibited positive selection. Finally, one of the two “neutral” markers used by [103] to compare with F_ST_-outliers was a fibrillar collagen gene (*FcolI/II/IIIf*) that ranked in the top 50 of our PSGs. *FcolI/II/IIIf* exhibited significant branch-sites tests in three species (*S. intermedius*, *S. nudus* and *H. pulcherrimus*) but not in *S. purpuratus*. The limited overlap between targets of selection identified in these F_ST_-outlier studies and our study suggests that the connection between selection at historical and contemporary time scales at most genes is weak, possibly because of the idiosyncratic linkage associations between SNPs and sites under selection [106] or the unpredictable nature of adaptive evolution.

## Conclusions

In summary, our study documented strong signals of positive selection across the genomes of nine sea urchin taxa inhabiting the north Pacific and Atlantic regions. Our reference-based alignment methods identified and excluded artifacts found in previous studies and were validated by independent Sanger sequencing. The candidate PSGs exhibited elevated rates of nonsynonymous substitutions and reduced heterozygosities, consistent with pervasive selective sweeps in species with large effective population sizes. The extensive number of positively selected genes was unexpected given the limited ecological divergence among species and the minor opportunities for sexual selection or sexual conflict. Although the selective agents responsible for the adaptive diversification remain largely unknown, we suggest that pathogens might be the primary drivers. Further study on the contribution of positive selection at these and other specific loci are critical for understanding the evolution of reproductive isolation among strongylocentrotid sea urchins.

## Additional files

Additional file 1: A Microsoft Word data file containing Supplementary **Tables S1–S13**. (DOC 92 kb)

Additional file 2: A Microsoft Word data file containing Supplementary **Figure S1–S27**. (DOC 5395 kb)

Additional file 3: Consensus alignment files. A compressed (gzip) tape archive (TAR) file containing 6250 consensus alignment files in FASTA format. (TGZ 14116 kb)

Additional file 4: Results of test for Positive Selection. A Microsoft Excel data file containing the summary of tests for positive selection at the 6520 single-copy orthologs. Also included are measures of codon bias (ENC and Nc’) and GC content. (XLS 3051 kb)

## Abbreviations

BAM: Binary Sequence/Alignment Map
*d*_*N*_: Rate of nonsynonymous substitution per nonsynonymous site
DNA: Deoxyribonucleic acid
*d*_*S*_: Rate of synonymous substitution per synonymous site
FDR: False Discovery Rate
GO: Gene Ontology
HG: Hypergeometric
IUPAC: International Union of Pure and Applied Chemistry
LRT: Likelihood Ratio Test
ML: Maximum Likelihood
NCBI: National Center for Biotechnology Information
NGS: Next Generation Sequencing
NLR: NACHT domain and leucine-rich repeat proteins
PAML: Phylogenetic Analysis by Maximum Likelihood
PCR: Polymerase Chain Reaction
PSC: Positively Selected Codon
PSG: Positive Selection Gene
SD: Standard Deviation
SNP: Single Nucleotide Polymorphism
SRA: Short Read Archive
SRCR: scavenger receptor cysteine-rich proteins
SSAHA2: Sequence Search and Alignment by Hashing Algorithm
TLR: Toll-like receptor
UCSC: University of California Santa Cruz

## References

[1] Lewontin R (1974). The genetic basis of evolutionary change.

[2] Kimura M (1983). The neutral theory of molecular evolution. Cambridge [Cambridgeshire].

[3] Lynch M (2007). The origins of genome architecture.

[4] Nielsen R (2005). Molecular signatures of natural selection. Annu Rev Genet.

[5] Oleksyk TK, Smith MW, O'Brien SJ (2010). Genome-wide scans for footprints of natural selection. Philos Trans R Soc Lond Ser B Biol Sci.

[6] Vitti JJ, Grossman SR, Sabeti PC (2013). Detecting natural selection in genomic data. Annu Rev Genet.

[7] Larracuente AM, Sackton TB, Greenberg AJ, Wong A, Singh ND, Sturgill D, Zhang Y, Oliver B, Clark AG (2008). Evolution of protein-coding genes in drosophila. Trends Genet.

[8] Kosiol C, Vinař T, da Fonseca RR, Hubisz MJ, Bustamante CD, Nielsen R, Siepel A (2008). Patterns of positive selection in six mammalian genomes. PLoS Genet.

[9] Arbiza L, Gronau I, Aksoy BA, Hubisz MJ, Gulko B, Keinan A, Siepel A (2013). Genome-wide inference of natural selection on human transcription factor binding sites. Nat Genet.

[10] Enard D, Messer PW, Petrov DA (2014). Genome-wide signals of positive selection in human evolution. Genome Res.

[11] Voight BF, Kudaravalli S, Wen X, Pritchard JK (2006). A map of recent positive selection in the human genome. PLoS Biol.

[12] Barreiro LB, Laval G, Quach H, Patin E, Quintana-Murci L (2008). Natural selection has driven population differentiation in modern humans. Nat Genet.

[13] Singh ND, Larracuente AM, Sackton TB, Clark AG (2009). Comparative genomics on the drosophila phylogenetic tree. Annu Rev Ecol Evol Syst.

[14] Fumagalli M, Sironi M, Pozzoli U, Ferrer-Admetlla A, Pattini L, Nielsen R (2011). Signatures of environmental genetic adaptation pinpoint pathogens as the main selective pressure through human evolution. PLoS Genet.

[15] Mathieson I, Lazaridis I, Rohland N, Mallick S, Patterson N, Roodenberg SA, Harney E, Stewardson K, Fernandes D, Novak M (2015). Genome-wide patterns of selection in 230 ancient Eurasians. Nature.

[16] Sattath S, Elyashiv E, Kolodny O, Rinott Y, Sella G (2011). Pervasive adaptive protein evolution apparent in diversity patterns around amino acid substitutions in Drosophila Simulans. PLoS Genet.

[17] Lee YC, Langley CH, Begun DJ (2014). Differential strengths of positive selection revealed by hitchhiking effects at small physical scales in Drosophila Melanogaster. Mol Biol Evol.

[18] Yang Z (2007). PAML 4: phylogenetic analysis by maximum likelihood. Mol Biol Evol.

[19] Anisimova M, Bielawski JP, Yang Z (2001). Accuracy and power of the likelihood ratio test in detecting adaptive molecular evolution. Mol Biol Evol.

[20] Yang Z, dos Reis M (2011). Statistical properties of the branch-site test of positive selection. Mol Biol Evol.

[21] Gharib WH, Robinson-Rechavi M (2013). The branch-site test of positive selection is surprisingly robust but lacks power under synonymous substitution saturation and variation in GC. Mol Biol Evol.

[22] Consortium DG (2007). Evolution of genes and genomes on the drosophila phylogeny. Nature.

[23] Ellegren H (2009). A selection model of molecular evolution incorporating the effective population size. Evolution.

[24] Mah SA, Swanson WJ, Vacquier VD (2005). Positive selection in the carbohydrate recognition domains of sea urchin sperm receptor for egg jelly (suREJ) proteins. Mol Biol Evol.

[25] Pujolar JM, Pogson GH (2011). Positive Darwinian selection in gamete recognition proteins of Strongylocentrotus sea urchins. Mol Ecol.

[26] Oliver TA, Garfield DA, Manier MK, Haygood R, Wray GA, Palumbi SR (2010). Whole-genome positive selection and habitat-driven evolution in a shallow and a deep-sea urchin. Genome Biol. Evol..

[27] Markow TA, O'Grady PM (2007). Drosophila biology in the genomic age. Genetics.

[28] Lawrence J (2013). Sea urchins: biology and ecology.

[29] Thompson JN (2013). Relentless evolution.

[30] Swanson WJ, Vacquier VD (2002). Reproductive protein evolution. Annu Rev Ecol Syst.

[31] Clark NL, Aagaard JE, Swanson WJ (2006). Evolution of reproductive proteins from animals and plants. In.

[32] Obbard DJ, Welch JJ, Kim KW, Jiggins FM (2009). Quantifying adaptive evolution in the drosophila immune system. PLoS Genet.

[33] Palumbi SR (2009). Speciation and the evolution of gamete recognition genes: pattern and process. Heredity (Edinb).

[34] Pearse J, Hines A: Long-term population dynamics of sea urchins in a central California kelp forest: rare recruitment and rapid decline. 1987.

[35] Scheibling RE, Stephenson R (1984). Mass mortality of *Strongylocentrotus droebachiensis* (Echinodermata: Echinoidea) off Nova Scotia, Canada. Mar Biol.

[36] Lessios HA (1984). Possible prezygotic reproductive isolation in sea urchins separated by the isthmus of Panama. Evolution.

[37] Britten RJ, Cetta A, Davidson EH (1978). The single-copy DNA sequence polymorphism of the sea urchin *Strongylocentrotus purpuratus*. Cell.

[38] Addison JA, Pogson GH (2009). Multiple gene genealogies reveal asymmetrical hybridization and introgression among strongylocentrotid sea urchins. Mol Ecol.

[39] Palumbi SR, Wilson AC (1990). Mitochondrial DNA diversity in the sea urchins *Strongylocentrotus purpuratus* and *S. droebachiensis*. Evolution.

[40] Sodergren E, Weinstock GM, Davidson EH, Cameron RA, Gibbs RA, Angerer RC, Angerer LM, Arnone MI, Burgess DR, Burke RD (2006). The genome of the sea urchin *Strongylocentrotus purpuratus*. Science.

[41] Cameron RA, Samanta M, Yuan A, He D, Davidson E (2009). SpBase: the sea urchin genome database and web site. Nucleic Acids Res.

[42] Tu Q, Cameron RA, Worley KC, Gibbs RA, Davidson EH (2012). Gene structure in the sea urchin *Strongylocentrotus purpuratus* based on transcriptome analysis. Genome Res.

[43] Lee YH (2003). Molecular phylogenies and divergence times of sea urchin species of Strongylocentrotidae, Echinoida. Mol Biol Evol.

[44] Kober K, Bernardi G (2013). Phylogenomics of strongylocentrotid sea urchins. BMC Evol Biol.

[45] Garrigan D, Hedrick PW (2003). Perspective: detecting adaptive molecular polymorphism: lessons from the MHC. Evolution.

[46] Kober KM, Pogson GH (2013). Genome-wide patterns of codon bias are shaped by natural selection in the purple sea urchin, *Strongylocentrotus purpuratus*. G3.

[47] Pogson GH, Mesa KA, Boutilier RG (1995). Genetic population structure and gene flow in the Atlantic cod Gadus Morhua: a comparison of allozyme and nuclear RFLP loci. Genetics.

[48] Karolchik D, Kuhn RM, Baertsch R, Barber GP, Clawson H, Diekhans M, Giardine B, Harte RA, Hinrichs AS, Hsu F *et al*: The UCSC Genome Browser Database: 2008 update. Nucleic Acids Res. 2008;36(Database issue):D773-9. Epub 2007 Dec 17. 10.1093/nar/gkm966 PMC2238835 18086701

[49] Junier T, Zdobnov EM (2010). The Newick utilities: high-throughput phylogenetic tree processing in the Unix shell. Bioinformatics.

[50] Ihaka R, Gentleman R (1996). R: a language for data analysis and graphics. J Comput Graph Stat.

[51] Li H, Handsaker B, Wysoker A, Fennell T, Ruan J, Homer N, Marth G, Abecasis G, Durbin R, Subgroup GPDP (2009). The sequence alignment/map format and SAMtools. Bioinformatics.

[52] Quinlan AR, Hall IM (2010). BEDTools: a flexible suite of utilities for comparing genomic features. Bioinformatics.

[53] Kent WJ, Sugnet CW, Furey TS, Roskin KM, Pringle TH, Zahler AM, Haussler D (2002). The human genome browser at UCSC. Genome Res.

[54] Ning Z, Cox AJ, Mullikin JC (2001). SSAHA: a fast search method for large DNA databases. Genome Res.

[55] Thanaraj TA, Clark F, Muilu J (2003). Conservation of human alternative splice events in mouse. Nucleic Acids Res.

[56] Palmieri N, Schlotterer C (2009). Mapping accuracy of short reads from massively parallel sequencing and the implications for quantitative expression profiling. PLoS One.

[57] Li H, Ruan J, Durbin R (2008). Mapping short DNA sequencing reads and calling variants using mapping quality scores. Genome Res.

[58] Li H, Handsaker B, Wysoker A, Fennell T, Ruan J, Homer N, Marth G, Abecasis G, Durbin R (2009). Genome project data processing S: the sequence alignment/map format and SAMtools. Bioinformatics.

[59] Markova-Raina P, Petrov D (2011). High sensitivity to aligner and high rate of false positives in the estimates of positive selection in the 12 drosophila genomes. Genome Res.

[60] Sousa V, Hey J (2013). Understanding the origin of species with genome-scale data: modelling gene flow. Nat Rev Genet.

[61] Fletcher W, Yang Z (2010). The effect of insertions, deletions, and alignment errors on the branch-site test of positive selection. Mol Biol Evol.

[62] Privman E, Penn O, Pupko T (2012). Improving the performance of positive selection inference by filtering unreliable alignment regions. Mol Biol Evol.

[63] Guindon S, Dufayard JF, Lefort V, Anisimova M, Hordijk W, Gascuel O (2010). New algorithms and methods to estimate maximum-likelihood phylogenies: assessing the performance of PhyML 3.0. Syst Biol.

[64] Tavaré S: Some probabilistic and statistical problems in the analysis of DNA sequences. In: American Mathematical Society: Lectures on Mathematics in the Life Sciences. Am Math Soc. 1986;17:57-86.

[65] Jordan G, Goldman N (2012). The effects of alignment error and alignment filtering on the sitewise detection of positive selection. Mol Biol Evol.

[66] Yang Z, Wong WS, Nielsen R (2005). Bayes empirical bayes inference of amino acid sites under positive selection. Mol Biol Evol.

[67] Strimmer K (2008). Fdrtool: a versatile R package for estimating local and tail area-based false discovery rates. Bioinformatics.

[68] Strimmer K (2008). A unified approach to false discovery rate estimation. BMC Bioinformatics.

[69] Zhang J, Nielsen R, Yang Z (2005). Evaluation of an improved branch-site likelihood method for detecting positive selection at the molecular level. Mol Biol Evol.

[70] Anisimova M, Yang Z (2007). Multiple hypothesis testing to detect lineages under positive selection that affects only a few sites. Mol Biol Evol.

[71] Lu A, Guindon S. Performance of standard and stochastic branch-site models for detecting positive selection among coding sequences. Mol Biol Evol. 2014;31(2):484-95. 10.1093/molbev/mst198 24132121

[72] Bisgrove BW, Andrews ME, Raff RA (1995). Evolution of the fibropellin gene family and patterns of fibropellin gene expression in sea urchin phylogeny. J Mol Evol.

[73] Bhattacharya G, Kalluri R, Orten DJ, Kimberling WJ, Cosgrove D (2004). A domain-specific usherin/collagen IV interaction may be required for stable integration into the basement membrane superstructure. J Cell Sci.

[74] Kunstner A, Wolf JB, Backstrom N, Whitney O, Balakrishnan CN, Day L, Edwards SV, Janes DE, Schlinger BA, Wilson RK (2010). Comparative genomics based on massive parallel transcriptome sequencing reveals patterns of substitution and selection across 10 bird species. Mol Ecol.

[75] Roux J, Privman E, Moretti S, Daub JT, Robinson-Rechavi M, Keller L (2014). Patterns of positive selection in seven ant genomes. Mol Biol Evol.

[76] Ellegren H (2008). Comparative genomics and the study of evolution by natural selection. Mol Ecol.

[77] Sackton TB, Lazzaro BP, Schlenke TA, Evans JD, Hultmark D, Clark AG (2007). Dynamic evolution of the innate immune system in drosophila. Nat Genet.

[78] Schlenke TA, Begun DJ (2003). Natural selection drives Drosophila immune system evolution. Genetics.

[79] Hibino T, Loza-Coll M, Messier C, Majeske AJ, Cohen AH, Terwilliger DP, Buckley KM, Brockton V, Nair SV, Berney K (2006). The immune gene repertoire encoded in the purple sea urchin genome. Dev Biol.

[80] Zhai W, Nielsen R, Slatkin M (2009). An investigation of the statistical power of neutrality tests based on comparative and population genetic data. Mol Biol Evol.

[81] Schneider A, Souvorov A, Sabath N, Landan G, Gonnet GH, Graur D (2009). Estimates of positive darwinian selection are inflated by errors in sequencing, annotation, and alignment. Genome Biol Evol.

[82] Kefalides NA, Alper R, Clark CC (1979). Biochemistry and metabolism of basement membranes. Int Rev Cytol.

[83] Abrahamson DR (1986). Recent studies on the structure and pathology of basement membranes. J Pathol.

[84] Vercellotti GM, McCarthy JB, Lindholm P, Peterson PK, Jacob HS, Furcht LT (1985). Extracellular matrix proteins (fibronectin, laminin, and type IV collagen) bind and aggregate bacteria. Am J Pathol.

[85] Westerlund B, Korhonen TK (1993). Bacterial proteins binding to the mammalian extracellular matrix. Mol Microbiol.

[86] Harrington DJ (1996). Bacterial collagenases and collagen-degrading enzymes and their potential role in human disease. Infect Immun.

[87] Singh B, Fleury C, Jalalvand F, Riesbeck K (2012). Human pathogens utilize host extracellular matrix proteins laminin and collagen for adhesion and invasion of the host. FEMS Microbiol Rev.

[88] Stewart PL, Nemerow GR (2007). Cell integrins: commonly used receptors for diverse viral pathogens. Trends Microbiol.

[89] Hauck CR, Agerer F, Muenzner P, Schmitter T (2006). Cellular adhesion molecules as targets for bacterial infection. Eur J Cell Biol.

[90] Elde NC, Malik HS (2009). The evolutionary conundrum of pathogen mimicry. Nat Rev Microbiol.

[91] Henry T, Gorvel JP, Meresse S (2006). Molecular motors hijacking by intracellular pathogens. Cell Microbiol.

[92] Valerio E, Chaves S, Tenreiro R (2010). Diversity and impact of prokaryotic toxins on aquatic environments: a review. Toxins (Basel).

[93] Collier RJ (1975). Diphtheria toxin: mode of action and structure. Bacteriol Rev.

[94] Jorgensen R, Purdy AE, Fieldhouse RJ, Kimber MS, Bartlett DH, Merrill AR (2008). Cholix toxin, a novel ADP-ribosylating factor from Vibrio cholerae. J Biol Chem.

[95] Lange A, Giller K, Hornig S, Martin-Eauclaire MF, Pongs O, Becker S, Baldus M (2006). Toxin-induced conformational changes in a potassium channel revealed by solid-state NMR. Nature.

[96] Haldane JBS (1949). Disease and evolution. Supplement to La Ricerca Scientifica.

[97] Barreiro LB, Quintana-Murci L (2010). From evolutionary genetics to human immunology: how selection shapes host defence genes. Nat Rev Genet.

[98] Harvell CD, Kim K, Burkholder JM, Colwell RR, Epstein PR, Grimes DJ, Hofmann EE, Lipp EK, Osterhaus AD, Overstreet RM (1999). Emerging marine diseases--climate links and anthropogenic factors. Science.

[99] Suttle CA (2005). Viruses in the sea. Nature.

[100] Pearse J, Costa D, Yellin M, Agegian C (1977). Localized mass mortality of red sea urchins, Strongylocentrotus Franciscanus, near Santa Cruz, California. Fish Bull.

[101] Beaumont MA, Balding DJ (2004). Identifying adaptive genetic divergence among populations from genome scans. Mol Ecol.

[102] Foll M, Gaggiotti O (2008). A genome-scan method to identify selected loci appropriate for both dominant and codominant markers: a Bayesian perspective. Genetics.

[103] Pespeni MH, Palumbi SR (2013). Signals of selection in outlier loci in a widely dispersing species across an environmental mosaic. Mol Ecol.

[104] Pespeni MH, Oliver TA, Manier MK, Palumbi SR (2010). Restriction site tiling analysis: accurate discovery and quantitative genotyping of genome-wide polymorphisms using nucleotide arrays. Genome Biol.

[105] Pespeni MH, Garfield DA, Manier MK, Palumbi SR (2012). Genome-wide polymorphisms show unexpected targets of natural selection. Proc Biol Sci.

[106] Bierne N, Welch J, Loire E, Bonhomme F, David P (2011). The coupling hypothesis: why genome scans may fail to map local adaptation genes. Mol Ecol.

