# Supplementary material for: Genome-wide signals of positive selection in strongylocentrotid sea urchins
Source: BMC Genomics. 2017 Jul 21;18:555. doi: 10.1186/s12864-017-3944-7 (PMC5521101; doi:10.1186/s12864-017-3944-7)
Supplement: Supplementary file 1 — A Microsoft Word data file containing Supplementary Tables S1–S13. (DOC 92 kb) [file 12864_2017_3944_MOESM1_ESM.doc]

Table S1. PCR primers used to amplify fragments for Sanger sequencing. The sequences for *CyclinD*, *SoxB2* and exon 21 of *Ebr1* were obtained by cloning one allele per individual as described in Addison and Pogson (2009) and Pujolar and Pogson (2011). For the remaining genes PCR products were sequenced directly from each individual.

_____________________________________________________________________________________

SPU Number Gene Name Sequence (5’ – 3’) Location

_____________________________________________________________________________________

SPU_000526 (Exon 14) Ebr1 Forward: TGCTCTGTGACTTGTGATACTGG Exon 14

Reverse: AAGACTCACGTCTCCGAATGA Exon 15

SPU_000526 (Exon 21) Ebr1 Forward: TGGTTCAAGCAGCAAGTCAGT Exon 22

Reverse: CTTTGGTGATTGTTCTGTGACC Exon 21

SPU_001452 Dachs Forward: GGCCCTCTCAGAGTTTGTCA Exon 19

Reverse: GATAGAACAGCTGCGGGAAG Exon 19

SPU_005955 NotchL Forward: CAGGCCATCAATCAACACAC Exon 7

Reverse: GGCGGAACATGCTTTGATAA Exon 7

SPU_007013 CyclinD Forward: ACCAATCAAATGGTAGCACGG 5’ UTR

Reverse: ACGTCAACAGGTTGTTCCTGG Intron 1

SPU_008159 Kcnk13 Forward: GATACAACCGTKGCGACAAA Exon 1

Reverse: CCATGGATAACCTACACATCRA 5’ UTR

SPU_009691 Plceta Forward: TGGCATTTCMGTCAGGTTCTT Exon 17

Reverse: TGATGCTTGGGAGATCAGC Exon 17

SPU_012948 Z50 Forward: CCTGGCAGGAAGTCAAGAGT Exon 6

Reverse: GGCAGATGCGACACTGATAA Exon 6

SPU_014051 Bindin Forward: AAGGGCAAGTCCTCGTAAGG Exon 4

Reverse: CCAATGTAGTGGTCGGTCCT Intron 4

SPU_025133 SoxB2 Forward: AAGTCTATAATCTGTCGCTCGC 5’ UTR

Reverse: AAAGGGAAAAGTTTCACCGG 3’ UTR

_____________________________________________________________________________________

Table S2. Examples of filters used to identify alignments containing paralogs.

____________________________________________________________________________________________

No. of Heterozygous Mutations^a^

No. of sites Mean ________________________________

Gene and Species with > 2 bases Coverage Low Bin Mid Bin High Bin

____________________________________________________________________________________________

**WITH PARALOGS**

SPU_007806

*S. droebachiensis* 0 97.0 41 44 42

*A. fragilis* 1 78.4 45 44 44

*S. pallidus* 5 28.9 83 56 81

*S. intermedius* 2 94.7 55 35 52

*H. pulcherrimus* 0 87.0 35 49 34

*S. nudus* 8 215.9 154 60 141

*S. franciscanus* 1 116.0 78 52 77

*P. depressus* 9 220.8 135 39 118

SPU_009673

*S. droebachiensis* 0 43.9 17 74 17

*A. fragilis*  0 50.7 5 26 5

*S. pallidus* 0 17.2 2 2 2

*S. intermedius* 2 69.9 126 56 123

*H. pulcherrimus* 0 54.9 2 26 2

*S. nudus* 2 103.6 78 74 74

*S. franciscanus* 1 78.4 101 37 100

*P. depressus* 0 54.7 5 10 5

SPU_012273

*S. droebachiensis* 0 28.3 1 0 1

*A. fragilis*  0 22.9 3 4 3

*S. pallidus* 0 17.2 9 4 9

*S. intermedius* 0 28.4 0 8 0

*H. pulcherrimus* 0 37.2 2 0 2

*S. nudus* 4 166.9 66 53 57

*S. franciscanus* 0 59.4 8 41 9

*P. depressus* 0 108.6 17 38 17

SPU_019899

*S. droebachiensis* 1 103.6 48 64 48

*A. fragilis*  6 147.2 120 71 107

*S. pallidus* 1 24.9 29 28 28

*S. intermedius* 1 146.7 91 68 91

*H. pulcherrimus* 0 29.4 34 11 37

*S. nudus* 0 48.5 5 16 5

*S. franciscanus* 0 42.1 0 2 0

*P. depressus* 0 54.2 12 34 12

**WITHOUT PARALOGS**

SPU_003412

*S. droebachiensis* 0 41.8 49 128 49

*A. fragilis* 0 50.8 2 13 3

*S. pallidus* 0 20.1 2 2 2

*S. intermedius* 0 49.8 7 99 8

*H. pulcherrimus* 0 62.9 9 14 9

*S. nudus* 0 53.4 4 12 4

*S. franciscanus* 0 46.6 9 24 9

*P. depressus* 0 42.7 6 16 6

SPU_004893

*S. droebachiensis* 0 56.7 5 38 5

*A. fragilis* 0 61.8 14 54 14

*S. pallidus* 0 15.5 22 39 25

*S. intermedius* 0 56.2 12 40 12

*H. pulcherrimus* 0 70.4 5 42 5

*S. nudus* 0 62.3 7 22 7

*S. franciscanus* 0 49.5 7 19 6

*P. depressus* 0 84.4 1 4 1

SPU_010384

*S. droebachiensis* 0 34.4 0 12 0

*A. fragilis* 0 50.3 1 4 1

*S. pallidus* 0 20.4 1 6 1

*S. intermedius* 0 48.6 2 14 2

*H. pulcherrimus* 0 63.2 0 6 0

*S. nudus* 0 64.8 1 12 1

*S. franciscanus* 0 48.7 1 10 1

*P. depressus* 0 59.4 0 2 0

SPU_022206

*S. droebachiensis* 0 51.9 2 18 2

*A. fragilis* 0 52.3 7 25 8

*S. pallidus* 0 18.9 8 10 8

*S. intermedius* 0 54.2 1 56 1

*H. pulcherrimus* 0 58.9 2 32 2

*S. nudus* 0 57.0 0 10 0

*S. franciscanus* 0 46.0 3 4 3

*P. depressus* 0 67.2 4 24 4

____________________________________________________________________________________________

^a^ Numbers of heterozygous mutations falling into low (0.125 < p < 0.375), mid (0.375 < p < 0.625) and high (0.625 < p < 0.875) allele frequency bins.

Table S3. Numbers of heterozygous mutations observed at the 6,250 single-copy orthologs.

____________________________________________________________________________________________________

Total No. of heterozygous mutations No. of heterozygous mutations per 100 bp

_______________________________ __________________________________

Species PSGs^a^ non-PSGs Total PSGs^a^ non-PSGs Total

____________________________________________________________________________________________________

*S. droebachiensis* 35,825 215,851 251,676 1.64 2.54 2.36

*A. fragilis* 32,293 194,566 226,859 1.48 2.29 2.13

*S. pallidus* 66,407 383,466 449,873 3.03 4.52 4.21

*S. intermedius* 40,963 238,920 279,883 1.87 2.82 2.62

*H. pulcherrimus* 27,207 154,663 181,870 1.24 1.82 1.70

*S. nudus* 16,989 94,897 111,886 0.776 1.12 1.05

*S. franciscanus* 18,732 110,952 129,684 0.856 1.31 1.21

*P. depressus* 13,704 76,621 90,325 0.626 0.903 0.846

____________________________________________________________________________________________________

^a^ Positively Selected Genes

Table S4. Numbers of codons filtered by PAML because of missing data or the presence of ambiguous sites.

___________________________________________________________________________________

Numbers of codons filtered due to

Total No. of ___________________________________

Grouping and Species Codons Filtered Missing data (%) Ambiguous sites (%)

___________________________________________________________________________________

1. Positively Selected Genes (N = 1,008)

*S. droebachiensis* 32,565 25,373 (77.9) 7,192 (22.1)

*A. fragilis* 32,553 26,171 (80.4) 6,382 (19.6)

*S. pallidus* 47,079 36,680 (77.9) 10,399 (22.1)

*S. intermedius* 33,692 26,135 (77.6) 7,557 (22.4)

*H. pulcherrimus* 34,927 29,109 (83.3) 5,818 (16.7)

*S. nudus* 40,370 36,493 (90.4) 3,877 (9.6)

*S. franciscanus* 65,729 61,541 (93.6) 4,188 (6.4)

*P. depressus* 41,461 38,203 (92.1) 3,258 (7.9)

TOTALS 328,376 279,705 (85.2) 48,671 (14.8)

2. Non-Positively Selected Genes (N = 5,512)

*S. droebachiensis* 152,477 128,310 (84.2) 24,167 (15.8)

*A. fragilis* 149,942 128,474 (85.7) 21,468 (14.3)

*S. pallidus* 221,414 190,301 (85.9) 31,113 (14.1)

*S. intermedius* 161,808 137,779 (85.1) 24,029 (14.9)

*H. pulcherrimus* 164,349 146,010 (88.8) 18,339 (11.2)

*S. nudus* 187,207 174,445 (93.2) 12,762 (6.8)

*S. franciscanus* 309,271 295,271 (95.5) 14,000 (4.5)

*P. depressus* 200,291 189,159 (94.4) 11,132 (5.6)

TOTALS 1,546,759 1,389,749 (89.8) 157,010 (10.2)

3. All genes (N = 6,520)

*S. droebachiensis* 185,042 153,683 (83.1) 31,359 (16.9)

*A. fragilis* 182,495 154,645 (84.7) 27,850 (15.3)

*S. pallidus* 268,493 226,981 (84.5) 41,512 (15.5)

*S. intermedius* 195,500 163,914 (83.8) 31,586 (16.2)

*H. pulcherrimus* 199,276 175,119 (87.9) 24,157 (12.1)

*S. nudus* 227,577 210,938 (92.7) 16,639 (7.3)

*S. franciscanus* 375,000 356,812 (95.1) 18,188 (4.9)

*P. depressus* 241,752 227,362 (94.0) 14,390 (6.0)

TOTALS 1,875,135 1,669,454 (89.0) 205,681 (11.0)

____________________________________________________________________________________

Table S5. Counts of positively selected sites (PSSs) and sites not exhibiting positive selection (non-PSSs) within 3, 5, or 7 amino acids of an exon border at the 1,008 genes exhibiting positive selection. For all three windows there is a significant under-representation of positively selected codons, which is opposite to that expected by alignment error.

_____________________________________________________________________

Distance from Site Within Outside Exact Test P-value

exon border Class Region Region

_____________________________________________________________________

3 amino acids PSS 176 2807 2.35e-06

Non-PSS 168520 1889995

5 amino acids PSS 349 2634 0.0018

Non-PSS 280811 1777704

7 amino acids PSS 505 2478 0.0024

Non-PSS 393119 1665396

_____________________________________________________________________

Table S6. Counts of positively selected sites (PSSs) and sites not exhibiting positive selection (non-PSSs) within 3, 5, or 7 amino acids of missing data regions at the 1,008 genes exhibiting positive selection. For all three windows there is a significant under-representation of positively selected codons, which is opposite to that expected by alignment error.

_____________________________________________________________________

Distance from Site Within Outside Exact Test P-value

missing data region Class Region Region

_____________________________________________________________________

3 amino acids PSS 151 2832 2.20e-16

Non-PSS 238511 1820005

5 amino acids PSS 249 2734 2.20e-16

Non-PSS 279014 1779502

7 amino acids PSS 319 2664 4.91e-13

Non-PSS 314202 1744314

_____________________________________________________________________

Table S7. Comparison of kappa, codon usage, and GC content of candidate positively selected genes (PSGs) and those not exhibiting positive selection (non-PSGs).

______________________________________________________________________________

Mean No. Mean Mean Mean Mean Mean Mean Mean

Category N of codons kappa ENC^a^ Nc’^b^ GC^c^ GC_3_^d^ GC_I_^e^ GC_F_^f^

______________________________________________________________________________

PSGs 1,008 502.4 2.28 55.1 51.6 0.488 0.503 0.352 0.361

Non-PSGs 5,512 341.8 2.85 55.1 51.3 0.487 0.510 0.351 0.364

TOTAL 6,520 366.6 2.76 55.1 51.3 0.487 0.509 0.351 0.363

______________________________________________________________________________

^a^ Mean effective number of codons (Wright 1980)

^b^ Novembre’s (2002) effective number of codons corrected for background GC content.

^c^ Mean GC content of protein-coding sequences.

^d^ Mean GC content of third codon positions.

^e^ Mean GC intron content. Due to the presence of genes lacking introns, the sample sizes for the PSGs and non-PSGs are 916 and 5,062, respectively.

^f^ Mean GC content of flanking regions (1,000 bp upstream and downstream of start and stop codons, respectively). Due to missing data, the sample sizes for the PSGs and non-PSGs are 1006 and 5,503, respectively.

Table S8. Comparison of hypothetical proteins identified in candidate positively selected genes (PSGs) and those not exhibiting positive selection (non-PSGs).

___________________________________________________________________________

No. of No. of Mean No. Mean Mean Mean Mean

Category Genes base pairs of codons LRT Score^a^ *d*_N_ *d*_S_ *d*_N_/*d*_S_

___________________________________________________________________________

PSGs 158 363,300 494.3 13.72 0.1184 0.316 0.405

Non-PSGs 552 964,707 378.5 1.35 0.0758 0.335 0.247

TOTAL 710 1,328,007 404.3 4.30 0.0853 0.331 0.282

___________________________________________________________________________

^a^ Mean Likelihood Ratio Test Score comparing PAML models M7 and M8.

Table S9. Comparison of kappa, codon usage, and GC content of hypothetical proteins identified in candidate positively selected genes (PSGs) and those not exhibiting positive selection (non-PSGs).

______________________________________________________________________________

Mean No. Mean Mean Mean Mean Mean Mean Mean

Category N of codons kappa ENC^a^ Nc’^b^ GC^c^ GC_3_^d^ GC_I_^e^ GC_F_^f^

______________________________________________________________________________

PSGs 158 494.3 2.30 55.3 54.1 0.485 0.461 0.352 0.359

Non-PSGs 552 378.5 2.32 55.6 52.6 0.489 0.498 0.351 0.360

TOTAL 710 404.3 2.31 55.5 53.0 0.488 0.490 0.351 0.359

______________________________________________________________________________

^a^ Mean effective number of codons (Wright 1980)

^b^ Novembre’s (2002) effective number of codons corrected for background GC content.

^c^ Mean GC content of protein-coding sequences.

^d^ Mean GC content of third codon positions.

^e^ Mean GC intron content. Due to the presence of genes lacking introns, the sample sizes for the PSGs and non-PSGs are 136 and 467, respectively.

^f^ Mean GC content of flanking regions (1,000 bp upstream and downstream of start and stop codons, respectively). Due to missing data, the sample size for the non-PSGs is 550.

Table S10. Complete list of enrichment tests for genes experiencing positive selection using the Tu *et al.* (2012) custom sea urchin Gene Ontology (GO) categories.

___________________________________________________________________________________________________________

Number of genes

________________________ Fold

Category Subcategory Tested PSG^a^ E[PSG]^b^ Enrichment^c^ *P*-value^d^

___________________________________________________________________________________________________________

Adhesion 115 49 17.8 2.8 <0.0001

Adhesion_ECMCollagen 15 10 2.3 4.3 <0.001

Adhesion_ECMFibropellin 5 3 0.8 3.9 0.0632

Adhesion_ECMLaminin 3 1 0.5 2.2 0.5130

Adhesion_ECMNeural 13 5 2.0 2.5 0.0690

Adhesion_ECMOther 11 4 1.7 2.4 0.1125

Adhesion_ECMProteoglycan 22 5 3.4 1.5 0.3163

Adhesion_ECMReceptorCadherin 14 8 2.2 3.7 0.0013

Adhesion_ECMReceptorGPCR 11 4 1.7 2.4 0.1226

Adhesion_ECMReceptorIgFN3 21 9 3.2 2.8 0.0056

Adhesion_ECMReceptorIntegrin 4 1 0.6 1.6 0.5896

Adhesion_ECMReceptorLRR 5 1 0.8 1.3 0.6488

Apoptosis 12 1 1.9 0.5 0.8986

Biomineralization 24 8 3.7 2.2 0.0410

Biomineralization_CarbonicAnhydrase 3 1 0.5 2.2 0.5047

Biomineralization_Collagen 6 4 0.9 4.3 0.0168

Biomineralization_MSP130 6 2 0.9 2.2 0.3179

Biomineralization_Other 1 1 0.2 6.5 0.2917

CalciumToolkit 106 22 16.4 1.3 0.1497

CellCycle 42 4 6.5 0.3 0.9356

Cytoskeleton 68 11 10.5 1.0 0.5915

Cytoskeleton_Actin 14 3 2.2 1.4 0.4669

Cytoskeleton_Dynein 10 2 1.5 1.3 0.5663

Cytoskeleton_MicrotubuleAssociated 15 2 2.3 0.9 0.7723

Cytoskeleton_Myosin 11 3 1.7 1.8 0.3161

Cytoskeleton_Tubulin 4 1 0.6 1.6 0.5904

Defensome 151 21 23.3 0.9 0.8358

Defensome_Antioxidant 9 2 1.4 1.4 0.5069

Defensome_BiotransformationOxidative 46 4 7.1 0.6 0.9622

Defensome_HSP 12 1 1.9 0.5 0.9020

Defensome_MetalDetoxification 3 1 0.5 2.2 0.5133

Defensome_Sensor 12 2 1.8 1.1 0.6535

Defensome_TransporterABC 19 6 2.9 2.0 0.0928

Defensome_TransporterIon 30 5 4.6 1.1 0.5918

EggActivation 77 15 11.9 1.3 0.2957

GermLineDeterminant 6 1 0.9 1.1 0.7176

GPCRRhodopsin 268 40 41.4 1.0 0.7785

GTPase 111 13 17.2 0.8 0.9393

GTPase_Galpha 7 1 1.1 0.9 0.7586

GTPase_Ras 55 5 8.5 0.6 0.9636

GTPase_RhoRegulator 36 6 5.6 1.1 0.5837

GTPase_Translational 8 1 1.2 0.8 0.8035

Histone 9 1 1.4 0.7 0.8323

Histone_RepcaptionIndependent 5 1 0.8 1.3 0.6610

Immunity 123 26 19.0 1.4 0.1088

Immunity_Coagulation 2 1 0.3 3.2 0.4135

Immunity_Effector 15 4 2.3 1.7 0.2610

Immunity_ReceptorNLR 3 1 0.5 2.2 0.5080

Immunity_ReceptorScavenger 21 7 3.2 2.2 0.0566

Immunity_ReceptorTLR 5 2 0.8 2.6 0.2591

Immunity_Signal 37 8 5.7 1.4 0.2730

Immunity_TF 33 4 5.1 0.8 0.8410

Kinase 81 17 11.8 1.4 0.1764

Kinase_AGC 10 3 1.5 1.9 0.2673

Kinase_Atypical 5 2 0.8 2.6 0.2557

Kinase_CAMK 15 3 2.3 1.3 0.5025

Kinase_CMGC 16 3 2.5 1.2 0.5457

Kinase_Other 10 2 1.5 1.3 0.5632

Kinase_TK 10 3 1.5 1.9 0.2653

Kinase_TKL 4 1 0.6 1.6 0.5958

Metabolism 794 116 122.8 0.9 0.9260

Metabolism_AminoAcid 119 19 18.4 1.0 0.6003

Metabolism_Carbohydrate 178 24 27.5 0.9 0.8861

Metabolism_Coenzyme 26 4 4.0 1.0 0.6658

Metabolism_Energy 106 14 16.4 0.9 0.8524

Metabolism_InorganicIon 107 27 16.5 1.6 0.0158

Metabolism_Lipid 127 15 19.6 0.8 0.9433

Metabolism_Nucleotide 54 4 8.3 0.5 0.9855

Metabolism_SecondaryMetabolites 90 9 13.9 0.6 0.9725

Metalloprotease 84 20 13.0 1.5 0.0599

Nervous 341 50 52.7 0.9 0.8304

Nervous_Chemosensory 176 28 27.2 1.0 0.6078

Nervous_GrowthFactorRelated 6 1 0.9 1.1 0.7129

Nervous_NeuropeptideRelated 28 3 4.3 0.7 0.8790

Nervous_NeurotransmitterRelated 37 8 5.7 1.4 0.2778

Nervous_Placode 9 1 1.4 0.7 0.8309

Nervous_Retinal 16 3 2.5 1.2 0.5429

Nervous_SynapticVesicleRelease 17 2 2.6 0.8 0.8218

Nervous_TF 52 2 8.0 0.2 0.9993

Nervous_VGIC 11 3 1.7 1.8 0.3166

Oogenesis 14 4 2.2 1.8 0.2248

Phosphatase 41 4 6.3 0.6 0.9316

Phosphatase_SerineThreonine 18 2 2.8 0.7 0.8406

Phosphatase_Tyrosine 23 2 3.6 0.6 0.9226

Signaling 104 16 16.1 1.0 0.6719

Signaling_Hedgehog 19 1 2.9 0.3 0.9736

Signaling_Notch 10 4 1.5 2.6 0.0947

Signaling_RTK 19 5 2.9 1.7 0.2232

Signaling_TGFB 46 6 7.1 0.8 0.8076

Signaling_WntFzd 13 1 2.0 0.5 0.9205

TF 230 24 35.6 0.7 0.9970

TF_bHLH 31 2 4.8 0.4 0.9763

TF_bzip 12 1 1.9 0.5 0.9037

TF_Ets 7 1 1.1 0.9 0.7655

TF_Homeo 73 12 11.3 1.1 0.5793

TF_NR 22 1 3.4 0.3 0.9843

TF_Other 28 5 4.3 1.2 0.5376

TF_ZNF 26 2 4.0 0.5 0.9505

TranslationFactor 20 3 3.1 1.0 0.6948

ZNF 119 11 18.4 0.6 0.9914

___________________________________________________________________________________________________________

^a^ Positively Selected Gene

^b^ Expected number of PSGs

^c^ Fold enrichment of observed PSGs to E[PSGs]

^d^ Empirical *P*-values were determined by the hypergeometric with 10,000 re-samplings from a total of 6,520 genes tested and 1,008 identified as PSGs.

Table S11. Significant enrichment of GO codes from the branch-sites tests.

_____________________________________________________________________________________________________________________

Species

__________________________________________________________

Category Description Sdro Afra Spal Sint Spur Hpul Snud Sfra Pdep

_____________________________________________________________________________________________________________________

**Molecular Function**

GO:0000166 Nucleotide binding 1 1 1 1 1 1 1

GO:0000287 Magnesium ion binding 1

GO:0003676 Nucleic acid binding 1 1 1 1 1 1 1 1

GO:0003677 DNA binding 1 1 1 1

GO:0003700 Sequence-specific DNA binding

transcription factor activity 1 1 1 1 1

GO:0003723 RNA binding 1

GO:0003824 Catalytic activity 1 1 1 1 1 1 1 1

GO:0003924 GTPase activity 1 1 1

GO:0004180 Carboxypeptidase activity 1 1 1

GO:0004181 Metallocarboxypeptidase activity 1 1 1

GO:0004222 Metalloendopeptidase activity 1 1

GO:0004252 Serine-type endopeptidase activity 1 1 1

GO:0004672 Protein kinase activity 1 1 1 1 1

GO:0004702 Receptor signaling protein serine/threonine

kinase activity 1

GO:0004713 Protein tyrosine kinase activity 1 1 1 1

GO:0004867 Serine-type endopeptidase inhibitor activity 1

GO:0004930 G-protein coupled receptor activity 1

GO:0005044 Scavenger receptor activity 1 1 1

GO:0005198 Structural molecule activity 1 1

GO:0005201 Extracellular matrix structural constituent 1

GO:0005215 Transporter activity 1 1 1

GO:0005216 Ion channel activity 1 1 1

GO:0005249 Voltage-gated potassium channel activity 1

GO:0005267 Potassium channel activity 1

GO:0005328 Neurotransmittersodium symporter activity 1

GO:0005488 Binding 1 1 1 1 1 1 1 1

GO:0005509 Calcium ion binding 1 1 1 1 1 1 1

GO:0005515 Protein binding 1 1 1 1 1 1 1 1 1

GO:0005524 ATP binding 1 1 1 1 1 1 1 1 1

GO:0005525 GTP binding 1 1 1 1

GO:0008146 Sulfotransferase activity 1

GO:0008234 Cysteine-type peptidase activity 1

GO:0008237 Metallopeptidase activity 1 1 1

GO:0008270 Zinc ion binding 1 1 1 1 1 1 1 1 1

GO:0008378 Galactosyltransferase activity 1 1

GO:0008484 Sulfuric ester hydrolase activity 1

GO:0009055 Electron carrier activity 1

GO:0015171 Amino acid transmembrane transporter activity 1

GO:0016491 Oxidoreductase activity 1

GO:0016787 Hydrolase activity 1

GO:0016849 Phosphorus-oxygen lyase activity 1

GO:0016881 Acid-amino acid ligase activity 1

GO:0016887 ATPase activity 1

GO:0017111 Nucleoside-triphosphatase activity 1

GO:0020037 Heme bonding 1

GO:0030170 Pyridoxal phosphate binding 1 1

GO:0031072 Heat shock protein binding 1

GO:0042802 Identical protein binding 1 1

GO:0043169 Cation binding 1

GO:0043565 Sequence-specific DNA binding 1 1 1

GO:0046872 Metal ion binding 1

GO:0051082 Unfolded protein binding 1

**Cellular Component**

GO:0005581 Collagen 1

GO:0005622 Intracellular 1 1 1 1 1 1 1 1

GO:0005634 Nucleus 1 1 1 1 1 1 1 1

GO:0005737 Cytoplasm 2 2 2

GO:0005764 Lysosome 1 1

GO:0005874 Microtubule 1

GO:0016020 Membrane 1 1 1 1 1 1 1 1 1

GO:0016021 Integral to membrane 1 1 1 1 1 1 1 1 1

GO:0043234 Protein complex 1

**Biological Process**

GO:0003333 Amino acid transmembrane transport 1

GO:0005975 Carbohydrate metabolic process 1 1 1 1 1

GO:0006096 Glycolysis 1

GO:0006184 GTP catabolic process 1

GO:0006281 DNA Repair 1

GO:0006457 Protein folding 1

GO:0006468 Protein phosphorylation 1 1 1 1 1

GO:0006486 Protein glycosylation 1 1 1

GO:0006508 Proteolysis 1 1 1 1 1 1

GO:0006520 Cellular amino acid metabolic process 1

GO:0006629 Lipid metabolic process 1 1 1 1

GO:0006810 Transport 1 1 1 1

GO:0006811 Ion transport 1 1 1

GO:0006812 Cation transport 1

GO:0006813 Potassium ion transport 1

GO:0006836 Neurotransmitter transport 1

GO:0006886 Intracellular protein transport 1 1

GO:0006913 Nucleocytoplasmic transport 1

GO:0007017 Microtubule-based process 1

GO:0007018 Microtubule-based movement 1 1

GO:0007154 Cell communication 1

GO:0007155 Cell Adhesion 1

GO:0007156 Homophilic cell adhesion 1 1 1

GO:0007165 Signal transduction 1 1

GO:0007178 Transmembrane receptor protein serine/

threonine kinase signaling pathway 1

GO:0007186 G-protein coupled receptor signaling pathway 1 1 1 1 1 1 1

GO:0007264 Small GTPase mediated signal transduction 1

GO:0008152 Metabolic process 1 1 1 1 1 1

GO:0009058 Biosynthetic process 1 1

GO:0009190 Cyclic nucleotide biosynthetic process 1

GO:0015031 Protein transport 1 1 1

GO:0030154 Cell differentiation 1

GO:0035556 Intracellular signal transduction 1 1

GO:0043086 Negative regulation of catalytic activity 1 1

GO:0046034 ATP metabolic process 1

GO:0051258 Protein polymerization 1

GO:0055085 Transmembrane transport 1 1 1 1 1 1

GO:0055114 Oxidation-reduction process 1 1 1 1 1 1 1

GO:0071805 Potassium ion transport process 1

_____________________________________________________________________________________________________________________

Sdro = *Strongylocentrotus droebachiensis*, Afra = *Allocentrotus fragilis*, Spal = *S. pallidus*, Sint = *S. intermedius*, Spur = *S. purpuratus*, Hpul = *Hemicentrotus pulcherrimus*, Snud = *S. nudus*, Sfra = *S. franciscanus*, Pdep = *Pseudocentrotus depressus*.

Table S12. Effect of retaining codons containing heterozygous sites on the signals of positive selection in the top 25 positively selected genes (PSGs). Retaining more codons in the tests for positive selection resulted in higher d_N_/d_S_ ratios (18 of 25), LRT scores (17 of 25), and the numbers of positively selected sites (19 of 25).

______________________________________________________________________________________________________

Codons with heterozygous sites removed^a^ Codons with heterozygous sites retained^b^

________________________________________ _________________________________________

No. of LRT No. of No. of LRT No. of

SPU Gene Codons *d*_N_/*d*_S_ Score^c^ PSSs^d^ Codons *d*_N_/*d*_S_ Score^c^ PSSs^d^

______________________________________________________________________________________________________

SPU_003768 1183 0.459 233.04 77 1279 0.433 219.75 84

SPU_010829 800 0.322 102.62 20 820 0.331 114.55 23

SPU_008159 405 0.378 91.68 11 422 0.427 96.49 13

SPU_018532 439 0.873 79.67 38 548 1.011 123.73 46

SPU_006534 691 0.473 66.79 12 783 0.539 63.64 13

SPU_008462 684 0.710 61.85 21 808 0.766 79.83 28

SPU_006645 184 0.914 61.38 12 190 0.953 67.43 14

SPU_000526 1428 0.357 59.84 9 1529 0.358 57.52 8

SPU_009154 1017 0.629 58.28 19 1150 0.599 62.21 19

SPU_003671 761 0.330 55.58 13 990 0.414 88.25 15

SPU_018517 424 0.815 54.02 16 492 0.976 117.80 23

SPU_002551 357 0.468 53.88 6 420 0.492 45.59 6

SPU_016836 1709 0.408 53.41 7 1839 0.421 50.32 7

SPU_005187 384 0.400 51.59 5 432 0.361 52.62 6

SPU_003825 152 0.354 51.20 4 155 0.331 51.75 4

SPU_022116 270 0.403 50.87 7 329 0.409 53.23 13

SPU_003110 132 0.490 48.39 6 135 0.552 51.79 8

SPU_024408 450 0.601 46.85 13 569 0.544 40.88 18

SPU_015864 305 0.531 45.51 4 361 0.573 59.70 6

SPU_028613 824 0.333 44.96 9 895 0.351 67.74 12

SPU_000649 941 0.481 44.32 11 1187 0.476 56.30 23

SPU_007625 378 0.473 43.12 18 395 0.478 37.08 18

SPU_021274 805 0.444 39.92 14 909 0.471 48.79 17

SPU_019495 696 0.178 39.91 5 914 0.235 33.88 8

SPU_010116 1398 0.714 39.17 6 1782 0.700 46.68 16

_______________________________________________________________________________________________________

^a^ Codons containing heterozygous sites were filtered from the data by PAML.

^b^ Codons containing heterozygous sites were retained in the data using the observed gene tree and maximum parsimony criteria. If a mutation at a heterozygous site matched the ancestral state, the derived mutation was removed. If neither mutation at a heterozygous site matched the ancestral state, one base was randomly chosen without knowing if it caused a synonymous or a nonsynonymous change.If a heterozygous site contained two nucleotides that were different from those present in all other species, then one nucleotide was randomly chosen without knowing if it resulted in a synonymous or nonsynonymous change.

^c^ Likelihood Ratio Test Score comparing PAML models M7 and M8.

^d^ No. of Positively Selected Sites (codons) with Bayes Empirical Bayes posterior probabilities > 0.95.

Table S13. Effect of retaining codons containing heterozygous sites on the signals of positive selection at 25 genes just below the empirical q-value threshold. Retaining more codons in the tests for positive selection resulted in higher d_N_/d_S_ ratios (11 of 25), LRT scores (9 of 25), and the numbers of positively selected sites (10 of 25).

______________________________________________________________________________________________________

Codons with heterozygous sites removed^a^ Codons with heterozygous sites retained^b^

________________________________________ _________________________________________

No. of LRT No. of No. of LRT No. of

SPU Gene Codons *d*_N_/*d*_S_ Score^c^ PSSs^d^ Codons *d*_N_/*d*_S_ Score^c^ PSSs^d^

______________________________________________________________________________________________________

SPU_009467 182 0.210 6.26 1 264 0.195 3.28 0

SPU_011429 105 0.300 6.24 1 130 0.289 6.05 1

SPU_009705 467 0.204 6.24 0 795 0.225 5.92 0

SPU_014555 359 0.362 6.23 2 391 0.392 7.57 2

SPU_024085 320 0.350 6.22 1 373 0.358 6.16 1

SPU_000228 415 0.280 6.20 1 511 0.242 5.47 1

SPU_014690 471 0.385 6.20 2 556 0.347 1.50 0

SPU_020799 333 0.271 6.20 2 432 0.359 18.29 5

SPU_004906 321 0.364 6.19 1 421 0.400 6.74 1

SPU_013656 320 0.125 6.19 3 395 0.093 3.04 2

SPU_011727 346 0.240 6.18 2 406 0.300 11.36 5

SPU_011893 270 0.462 6.18 0 358 0.553 9.93 1

SPU_006221 315 0.067 6.18 0 357 0.061 7.06 1

SPU_017798 542 0.108 6.17 1 615 0.096 7.01 2

SPU_013769 429 0.141 6.17 1 527 0.169 9.81 4

SPU_004993 754 0.146 6.16 0 1054 0.115 2.20 0

SPU_012790 100 0.436 6.15 1 133 0.355 3.93 1

SPU_024008 418 0.277 6.14 1 482 0.297 7.63 2

SPU_019691 368 0.059 6.14 0 420 0.051 4.01 0

SPU_017088 130 0.339 6.13 0 173 0.382 4.26 0

SPU_017688 217 0.223 6.13 1 247 0.228 5.80 2

SPU_017018 366 0.316 6.13 1 460 0.275 5.08 2

SPU_016737 273 0.402 6.13 0 444 0.311 2.59 0

SPU_020330 1306 0.385 6.12 1 1797 0.378 9.30 2

SPU_018805 463 0.041 6.11 0 475 0.040 6.27 0

_______________________________________________________________________________________________________

^a^ Codons containing heterozygous sites were filtered from the data by PAML.

^b^ Codons containing heterozygous sites were retained in the data using the observed gene tree and maximum parsimony criteria. If a mutation at a heterozygous site matched the ancestral state, the derived mutation was removed. If neither mutation at a heterozygous site matched the ancestral state, one base was randomly chosen without knowing if it caused a synonymous or a nonsynonymous change.If a heterozygous site contained two nucleotides that were different from those present in all other species, then one nucleotide was randomly chosen without knowing if it resulted in a synonymous or nonsynonymous change.

^c^ Likelihood Ratio Test Score comparing PAML models M7 and M8.

^d^ No. of Positively Selected Sites (codons) with Bayes Empirical Bayes posterior probabilities > 0.95.
