# Supplementary material for: Genome-wide signals of positive selection in strongylocentrotid sea urchins
Source: BMC Genomics. 2017 Jul 21;18:555. doi: 10.1186/s12864-017-3944-7 (PMC5521101; doi:10.1186/s12864-017-3944-7)
Supplement: Supplementary file 2 — A Microsoft Word data file containing Supplementary Figure S1–S27. (DOC 5395 kb) [file 12864_2017_3944_MOESM2_ESM.doc]

Figure S1. Screenshot of the UCSC Genome Browser displaying the sea urchin *Sp-Ef2* (SPU_010829) gene region on Scaffold 372 of the strPur4 (Spur_3.1) assembly. Assembly tracks show scale, scaffold and positions of the region, and gaps in the assembly. The gene model is provided by the SpBase Official Gene Set Build 7 and supported by alignments of *S. purpuratus* mRNA sequences from GenBank. Gene models depict exons as solid blocks connected by lines in introns with arrows showing the direction of transcription. Amino acid sites identified as positively selected by codeml (with Bayes Empirical Bayes posterior probabilities > 0.95) are identified as bar plots of the negative log10 of the probability that the *d*_N_/*d*_S_ ratio (i.e., ω-ratio) >1. Alignments of short reads to the region for each species are displayed as separate track for Illumina and 454 reads. CpG islands, GC percent, and predicted repeats in the region are displayed in the final tracks.

Figure S2. Relationships between the magnitude of positive selection and (a) mean *d*_N_, (b) mean *d*_S_, and (c) mean *d*_N_/*d*_S_. Genes were ranked by their likelihood ratio test (LRT) scores comparing PAML models M7 and M8 and grouped into bins of 100 loci (the 65^th^ bin contained 120 genes). The first 10 bins contain 99.2% of the candidate positive selected genes. Values presented represent the means ± one standard deviation of the genes in each bin.

Figure S3. Relationships between the magnitude of positive selection and (a) the mean number of codons, (b) the mean effective number of codons (ENC) and (c) the mean GC content. Genes were ranked by their likelihood ratio test (LRT) scores comparing PAML models M7 and M8 and grouped into bins of 100 loci (the 65^th^ bin contained 120 genes). The first 10 bins contain 99.2% of the candidate positive selected genes. Values presented represent the means ± one standard deviation of the genes in each bin.

**Reference**

P D S D I G K E I K K K A E I G E P F N C T V L S

*S. purpuratus* CCTGATAGTGATATCGGCAAAGAAATTAAAAAGAAGGCCGAAATAGGTGAACCATTTAATTGCACTGTCCTGTCA

**Illumina**

P D S D I G K E I K K K A E ***I*** G E ***P*** F N C T V L S

*S. purpuratus* CCTGATAGTGATATCGGCAAAGAAATTAAAAAGAAGGCCGAAATAGGTGAACCATTTAATTGCACTGTCCTGTCA

. . N . . . . . . Q . . H D N . . E . S . . . . .

*S. droebachiensis*6 CCTGATAATGATATCGGCAAAGAAATTCAAAAGAAGCAyGACAATGGTGAAGAATTTAGTTGCACTGTCCTGTCA

. . N . V . R . . Q R . . D Q N . N . S . . . . .

*S. intermedius*1 CCTGATAATGATGTCGGCAGAGAAATTCAAAGGAAGGCCGACCAGAATGAAAATTTTAGTTGCACTGTCCTGTCA

. . N . . . . . . Q R . . . N ? . Q . . . . . . .

*S. pallidus*20 CCTGATAATGACATCGGCAAAGAAATTCAAAGGAAGGCCGAAATAANTGAGCAATTTAATTGCACTGTCCTGTCA

. . N . . . . . . Q . . . K L D . S . . . . . . .

*A. fragilis*10 CCTGATAATGACATCGGCAAAGAAATTCAAAAGAAGGCCAAACTTGATGAAAGTTTTAATTGCACTGTCCTGTCA

. . N E . . R . . Q . . . D A D . S . S . . . . .

*H. pulcherrimus*5 CCTGATAATGAGATCGGCAGAGAAATTCAAAAGAAGGCAGACGCTGATGAATCTTTTAGTTGCACTGTCCTGTCA

. . . . V . . . . Q R . H D L D . T . M . . . . .

*S. franciscanus*4 CCTGATAGTGACGTCGGCAAAGAAATTCAAAGGAAGCACGACCTTGATGAAACTTTTATGTGCACTGTCCTGTCA

. . G E . . . . . Q . . H . R N . T . M . . . . .

*S. nudus*1 CCTGATGGTGAGATCGGCAAAGAAATTCAAAAGAAGCACGAACGGAATGAAACTTTTATGTGCACTGTCCTGTCA

. . N . . . . . . Q . . H D N . . S . M . . . . .

*P. depressus*5 CCCGATAACGATATCGGCAAAGAAATTCAAAAGAAGCATGACAACGGTGAATCTTTTATGTGCACTGTCCTGTCA

..*...***..**......*.......*...*....****.******...****....**...............

Figure S4. Alignment of Illumina data for a section of SPU_003110 (*Sp-Eif5A*) in exons 3 and 4 (the last five amino acids shown). Underlined sites were removed prior to tests for positive selection. Positively selected codons are italicized and shown in bold in the Illumina alignment. Two positively selected codons occur in this region without any gaps in the alignment. The region corresponds to amino acids 117–141 in SPU_003110.

**Reference**

G P I P N V F E M K G V K G D R G E P G P D G P Q

*S. purpuratus* GGTCCAATCCCTAACGTCTTTGAAATGAAGGGTGTGAAAGGAGACCGTGGTGAGCCTGGTCCCGATGGTCCTCAG

**Illumina**

G P I P ***N*** V F E M K G V ***K*** G ***D*** R G ***E*** P G ***P D*** G P ***Q***

*S. purpuratus* GGTCCAATCCCTAACGTCTTTGAAATGAAGGGTGTGAAAGGAGACCGTGGTGAGCCTGGTCCCGATGGTCCTCAG

. . . . . . . . . . . . T . E . . L . . . . . . .

*S. droebachiensis*6 GGTCCAATTCCTAATGTCTTTGAAATGAAGGGTGTGACCGGAGAGCGTGGTCTGCCTGGTCCCGATGGTCCTCAG

. . . . . . . . . . . . P . E . . L . . . . . . .

*S. intermedius*1 GGTCCAATTCCTAATGTCTTTGAAATGAAGGGTGTGCCCGGAGAGCGTGGTCTGCCTGGTCCCGATGGTCCTCAG

. . . . D . . . . . . . P . N . . L . . . . . . .

*S. pallidus*20 GGTCCAATTCCTGATGTCTTTGAAATGAAGGGTGTGCCCGGAAATCGTGGTCTGCCTGGTCCCGATGGTCCTCAG

. . . . . . . . . . . . T . E . . L . . . P . . .

*A. fragilis*10 GGTCCAATTCCTAATGTCTTTGAAATGAAGGGTGTGACCGGAGAGCGTGGTCTGCCTGGTCCCCCTGGTCCTCAG

. . . . . . . D . . . . . . E . . L . . . . . . .

*H. pulcherrimus*5 GGTCCAATTCCAAATGTCTTTGATATGAAGGGTGTGAAAGGAGAGCGTGGTCTGCCTGGTCCCGATGGTCCTCAG

. . . . . . . . . . . . . . . . . A . . A P . . .

*S. franciscanus*4 GGTCCAATTCCAAATGTCTTTGAAATGAAGGGTGTGAAGGGAGACCGTGGTGCGCCTGGTGCGCCTGGTCCTCAG

. . . . S . . . . . . . . . . . . L . . . . . . M

*S. nudus*1 GGCCCAATTCCATCTGTCTTTGAAATGAAGGGTGTGAAGGGAGACCGTGGTCTGCCTGGTCCCGATGGTCCTATG

. . . . D . . . . . . . . . . . . L . . S . . . P

*P. depressus*5 GGTCCCATTCCAGATGTCTTTGAAATGAAGGGTGTGAAAGGAGACCGTGGTCTGCCTGGTTCTGATGGTCCTCCC

..*..*..*..****........*............***...*.*......**.......*.***.......***

Figure S5. Alignment of Illumina data for a section of SPU_003768 (*3Apcol*) at the beginning of exon 36. Positively selected codons are italicized and shown in bold in the Illumina alignment. Seven positively selected codons occur in this exon without any gaps in the alignment. The region corresponds to amino acids 1167–1191 in SPU_003768.

**Reference**

K S A S D Q S L A A Y K T A V E I A G I E L T P T

*S. purpuratus* AAGTCGGCGTCCGATCAGAGTCTAGCAGCGTACAAAACGGCCGTAGAGATTGCAGGAATTGAGTTAACACCCACA

**Illumina**

K S A S D Q S L ***A*** A Y K ***T*** A V E I A ***G I*** E L T P T

*S. purpuratus* AAGTCGGCGTCCGATCAGAGTCTAGCAGCGTACAAAACGGCCGTAGAGATTGCAGGAATTGAGTTAACACCCACA

. . . . . E . . . . . . A . . . . . . L . . . . .

*S. droebachiensis*6 AAGTCGGCGTCTGATGAGAGTCTAGCAGCGTACAAAGCGGCCGTAGAAATTGCAGGACTTGAGTTAACACCCACA

. . . . . E . . . . . . . . . . . . A L . . . . .

*S. intermedius*1 AAGTCGGCGTCCGATGAGAGTCTAGCAGCGTACAAAACGGCCGTAGAAATTGCAGCACTTGAGTTAACACCCACA

. . . . . . . . E . . . A . . . . . . S . . . . .

*S. pallidus*20 AAGTCGGCGTCTGATCAGAGTCTAGAAGCGTACAAAGCGGCCGTAGAGATTGCAGGGTCTGAGTTAACACCCACA

. . . . . . . . . . . . M . . . . . A A . . . . .

*A. fragilis*10 AAGTCGGCGTCCGATCAGAGTCTAGCAGCGTACAAAATGGCCGTAGAGATTGCAGCAGCTGAGTTAACACCCACA

. . . . . H . . D . . . M . . D . . V V . . . . .

*H. pulcherrimus*5 AAGTCAGCCTCTGATCACAGTCTAGATGCCTACAAAATGGCCGTAGACATTGCAGTAGTTGAGTTAACACCCACA

. . . . . . . . . . . . Q . . D . . V V . . . . .

*S. franciscanus*4 AAGTCAGCCTCCGATCAGAGTCTAGCAGCrTACAAACAGGCCGTCGACATTGCAGTAGTTGAGTTAACACCCACA

. . . . . H . . . . . . A . . D . . . V . . . . .

*S. nudus*1 AAGTCAGCCTCTGATCACAGTCTAGCAGCGTACAAAGCGGCCGTCGACATTGCAGGAGTAGAGTTAACACCCACA

. . . . . . . . . . . . E . . . . . . S . . . . .

*P. depressus*5 AAGTCAGCCTCTGATCAGAGTCTAGCAGCGTACAAAGAGGCCGTCGAAATTGCAGGATCTGAGTTAACACCCACA

.....*..*..*...*.*.......**..*......**......*..*.......*****...............

Figure S6. Alignment of Illumina data for a section of SPU_003825 (*Sp-14-3-3e*) in exon 4. Underlined site was removed prior to tests for positive selection. Positively selected codons are italicized and shown in bold in the Illumina alignment. Four positively selected codons occur in this exon without any gaps in the alignment. The region presented represents amino acids 144–168 in SPU_003825.

**Reference**

T T E V L Y V A T D K S F E I N Q C I I S V T V D

*S. purpuratus* ACGACCGAGGTTCTTTATGTGGCTACGGACAAGTCTTTTGAAATCAACCAATGCATTATAAGCGTAACGGTCGAT

**Illumina**

T T E V L Y V A ***T*** D K S F ***E*** I N ***Q*** C I I S V T V D

*S. purpuratus* ACGACCGAGGTTCTTTATGTGGCTACGGACAAGTCTTTTGAAATCAACCAATGCATTATAAGCGTAACGGTCGAT

. . Q . . . . . S . M . . K . I E . . . . . . . N

*S. droebachiensis*6 ACGACCCAGGTTCTATATGTGGCTTCGGACATGTCTTTTAAAATCATCGAATGCATTATAAGCGTAACGGTCAAT

. . . . . . . . . . . . . . . . . . . . . . . . N

*S. intermedius*1 ACGACCGAGGTTCTTTATGTGGCTACGGACAAGTCTTTTGAAATCAACCAATGCATTATAAGCGTAACGGTCAAT

. . . . . . . . S . M . . Q . S E . . . . . . . N

*S. pallidus*20 ACGACCGAGGTTCTATATGTGGCTTCGGACATGTCTTTTCAAATCAGCGAATGCATTATAAGCGTAACGGTCAAT

. . . . . . . . . . M . . K . S E . . . . . . . N

*A. fragilis*10 ACGACCGAGGTTCTTTATGTGGCTACGGACATGTCTTTTAAAATTAGCGAATGCATTATAAGCGTAACGGTCAAT

. . . . . . . . . . M/V . . Q . R D . . . . . . . N

*H. pulcherrimus*5 ACGACCGAGGTTCTTTATGTGGCTACAGACrTGTCTTTTCAAATCAGAGATTGCATTATAAGCGTAACGGTCAAT

. . Q . . . . . A . M . . N . . . . . . . . . . N

*S. franciscanus*4 ACGACCCAGGTTCTATATGTGGCTGCAGACATGTCTTTTAATATCAACCAATGCATTATAAGCGTAACGGTCAAT

. . Q . . . . . A . M . . N . . L . . . . . . . N

*S. nudus*1 ACTACCCAGGTTCTTTATGTGGCTGCAGACATGTCCTTTAATATCAACCTATGCATTATAAGCGTAACGGTCAAT

. . Q . . . . . . . M . . N . . L . . . . . . . N

*P. depressus*5 ACGACCCAGGTTCTTTATGTkGCTACAGACATGTCTTTTAATATCAACCTATGCATTATAAGCGTAACGGTCAAT

..*...*.......*.....*...*.*...**...*...*.*..*.*****.....................*..

Figure S7. Alignment of Illumina data for a section of SPU_006534 (*Sp-Ebr1_5*) in exon 6. Underlined sites were removed prior to the tests for positive selection. Positively selected codons are italicized and shown in bold in the Illumina alignment. Three positively selected codons occur in this exon without any gaps in the alignment. The region presented corresponds to amino acids 403–427 in SPU_006534.

**Reference**

R F A K F V T F V N N F D E E D P K T F S G L D P

*S. purpuratus* CGCTTCGCCAAATTTGTGACGTTTGTGAACAATTTTGATGAGGAGGACCCTAAGACCTTCAGTGGACTTGATCCC

**Illumina**

R F ***A K*** F V ***T*** F V ***N*** N F D E E D P K T F ***S*** G L D P

*S. purpuratus* CGCTTCGCCAAATTTGTGACGTTTGTGAACAATTTTGATGAGGAGGACCCTAAGACCTTCAGTGGACTTGATCCC

. . S . . L . . . T K . . . . . . . . . N . . N .

*S. droebachiensis*6 CGCTTCAGTAAATTCCTGACGTTTGTGACAAAGTTTGATGAGGAGGACCCTAAGACCTTCAATGGACTTAATCCC

. . R N . L I . . T K . . . . . . . . . G . . N .

*S. intermedius*1 CGCTTCAGAAACTTCCTGATTTTTGTGACAAAGTTTGATGAGGAGGACCCTAAGACCTTCGGTGGACTTAATCCC

. . S . . L . . . T K . . . . . . . . . N . . N .

*S. pallidus*20 CGCTTCAGTAAATTCCTGACGTTTGTGACAAAGTTTGATGAGGAGGACCCTAAGACCTTCAATGGACTTAATCCC

. . G . . L . . . T K . . . . . . . . . A . . N .

*A. fragilis*10 CGCTTTGGTAAATTCCTGACGTTTGTGACAAAGTTTGATGAGGAGGACCCTAAGACCTTCGCTGGACTTAATCCC

. . S . . L . . . . . . . . . . . . . . N . V . .

*H. pulcherrimus*5 CGCTTCAGCAAATTCCTGACGTTTGTGAACAATTTTGATGAGGAGGACCCTAAGACCTTCAATGGAGTTGATCCC

. . K N . L Q . . G . . . . . N . . . . N . V . .

*S. franciscanus*4 CGCTTCAAGAATTTCCTGCAGTTTGTGGGCAATTTTGATGAGGAGAACCCkAAGACCTTCAACGGAGTTGATCCC

. . R S . L M . . S . . . . . N . . . . N . V . .

*S. nudus*1 CGCTTCAGGTCTTTCCTGATGTTTGTGAGCAATTTTGATGAGGAGAACCCTAAGACCTTCAATGGAGTTGATCCC

. . K N . L Q . . S . . . . . . . . . . . . V . .

*P. depressus*5 CGCTTCAAGAATTTCCTGCAGTTTGTGAGCAATTTTGATGAGGAGGACCCTAAGACCTTCAGTGGAGTTGATCCC

.....*******..**..***......***..*............*....*.........***...*..*.....

Figure S8. Alignment of Illumina data for a section of SPU_006645 (*Sp-Gdi1_1*) in exon 5. Underlined site was removed prior to tests for positive selection. Positively selected codons are italicized and shown in bold in the Illumina alignment. Five positively selected codons occur in this exon without any gaps in the alignment. The region shown corresponds to amino acids 140–164 in SPU_006645.

**Reference**

A P F Q F G S L I N F V D K I I G T Y S M N N S E R

*S. purpuratus* GCGCCGTTCCAATTTGGATCGCTAATCAATTTTGTGGACAAAATCATAGGTACATATTCTATGAATAATAGCGAGCGT

**Illumina**

A P F Q F G S L I ***N*** F V ***D*** K I I G T Y S M ***N*** N ***S*** E R

*S. purpuratus* GCGCCGTTCCAATTTGGATCGCTAATCAATTTTGTGGACAAAATCATAGGTACATATTCTATGAATAATAGCGAGCGT

. . . . . . A . . K V . N H . . . S . . . . . . . .

*S. droebachiensis*6 GCGCCGTTCCAATTTGGAGCGCTAATCAAAGTGGTAAACCATATCATAGGTTCATATTCTATGAATAATAGCGAGCGT

. . . . . . A . . K V . S H . V . S . . . R . . . .

*S. intermedius*1 GCGCCGTTCCAATTTGGAGCGCTAATCAAAGTGGTAAGCCATATCGTAGGTTCATATTCTATGAGAAATAGCGAGCGT

. . . . . . A . . R V . . H . I/M . S . . . K N/D . . .

*S. pallidus*20 GCGCCGTTCCAATTTGGAGCGCTAATCAGGGTGGTAGACCATATCATrGGTTCATATTCTATGAAArATAGCGAGCGT

. . . . . . . . . . . . . . . . . S . . . K S . . .

*A. fragilis*10 GCGCCGTTCCAATTTGGATCGCTAATCAATTTTGTGGACAAAATCATAGGTTCATATTCTATGAAGAGTAGCGAGCGT

. . . . . . A . . K V . A H . . D S . . . K T . . .

*H. pulcherrimus*5 GCACCGTTCCAATTTGGAGCGCTAATCAAGGTGGTAGCCCATATCATAGATTCATATTCTATGAAAACTAGCGAGCGT

. . . . . . . . . K V . G S . . . S . . L . Q . . .

*S. franciscanus*4 GCGCCGTTCCAATTTGGATCGCTAATCAAAGTGGTGGGCAGTATCATAGGTTCATATTCTTTGAATCAAAGCGAGCGT

. . . . . . . . . R V L . . . . . . . . L I H A . .

*S. nudus*1 GCGCCCTTCCAATTTGGATCGCTAATCAGGGTGTTGGACAAAATCATTGGTACATATTCTTTAATTCATGCCGAGCGT

. . . . . . . . . V L . G R F R Y S H . V I . L . .

*P. depressus*5 GCGCCGTTCCAATTTGGATCGCTAATCGTATTGGTGGGCAGATTCAGATATTCACATTCTGTTATTAATTTGGAGCGT

..*..*............*........****.**.***.****..*****.*..*.....*.*.********......

Figure S9. Alignment of Illumina data for a section of SPU_007625 (*Sp-Nmur2*) in exon 1. The two underlined sites were removed prior to testing for positive selection. Positively selected codons are italicized and shown in bold in the Illumina alignment. Four positively selected codons occur in this region of exon 1 without any gaps in the alignment. The amino acids shown correspond to positions 296–321 in SPU_007625.

**Reference**

D F K L R S R Y L I D K Y N F E A Q E S R K I W C

*S. purpuratus* GATTTCAAACTCCGTAGCCGTTATTTGATTGACAAATATAACTTTGAGGCCCAAGAATCCCGTAAGATCTGGTGC

**Illumina**

D ***F*** K ***L*** R S R Y L I D K Y ***N*** F E ***A Q*** E S R K I W C

*S. purpuratus* GATTTCAAACTCCGTAGCCGTTATTTGATTGACAAATATAACTTTGAGGCCCAAGAATCCCGTAAGATCTGGTGC

. P . A . . . . . . . . . G . D . T . . . . . . .

*S. droebachiensis*6 GATCCCAAAGCCCGTAGCCGTTATTTGATTGATAAATATGGCTTCGACGCCACAGAATCCCGTAAGATCTGGTGC

. . . T . . . . . V . . . G . . V . . . . . . . .

*S. intermedius*1 GATTTCAAAACCCGTAGCCGTTATTTGGTTGATAAATATGGCTTTGAAGTCCAAGAATCCCGTAAGATCTGGTGC

. P . A . T . . . . E . . G . D Q T . . . . . . .

*S. pallidus*20 GATCCCAAAGCCCGTACCCGTTATTTGATTGAAAAATATGGCTTCGACCAAACAGAATCCCGTAAGATCTGGTGC

. . . . . . . . . . E . . G . D H T . . . . . . .

*A. fragilis*10 GATTTCAAACTCCGTAGCCGTTATTTGATCGAGAAATATGGCTTCGACCACGTAGAATCsCGTAAGATCTGGTGC

E . . A . . . . . . . . . G . D S . . . . . . . .

*H. pulcherrimus*5 GAATTCAAAGCCCGTAGCCGTTATTTGATTGACAAATATGGCTTCGATAGCCAAGAATCCCGTAAGATCTGGTGC

. P . V . . . . . . . . . . . D G G . . . . . . .

*S. franciscanus*4 GACCCCAAAGTCCGTAGTCGTTATTTGATCGACAAATACAACTTCGATGGCGGAGAATCCCGTAAGATCTGGTGC

. P . V . . . . . . . . . . . D G T . . . . . . .

*S. nudus*1 GACCCCAAAGTCCGTAGTCGTTATTTGATCGACAAATACAACTTCGATGGCACTGAATCCCGTAAGATCTGGTGC

. P . V . . . . . . . . . K . D G T . . . . . . .

*P. depressus*5 GACCCCAAAGTCCGTAGTCGTTATTTGATCGACAAATACAAATTCGATGGCACAGAATCCCGTAAGATCTGGTGC

..***....**.....**.........*.*..*.....****..*..*******.....*...............

Figure S10. Alignment of Illumina data for a section of SPU_010829 (*Sp-Ef2*) in exon 14. Underlined site was removed prior to tests for positive selection. Positively selected codons are italicized and shown in bold in the Illumina alignment. Five positively selected codons occur in this region of exon 14 without any gaps in the alignment. The amino acids shown correspond to positions 670–694 of SPU_010829.

**Reference**

D E A V A F S L A T S I P V A G T P Y S S G F T A E

*S. purpuratus* GATGAAGCGGTGGCGTTCAGTCTAGCGACGTCGATACCCGTGGCAGGGACGCCGTACTCCTCCGGCTTTACCGCAGAA

**Illumina**

D E A V A F S L A T S I ***P V*** A G T P Y S S G F T A E

*S. purpuratus* GATGAAGCGGTGGCGTTCAGTCTAGCGACGTCGATACCCGTGGCAGGGACGCCGTACTCCTCCGGCTTTACCGCAGAA

. . . . . . . . . . P . . I T . . S . . . . . . . .

*S. droebachiensis*6 GATGAAGCGGTGGCGTTCAGTCTAGCGACGCCGATACCCATTACAGGGACGTCGTACTCCTCCGGCTTTACCGCAGAA

. . V . . . . . . L P M S M P . . Q . . . . . . . .

*S. intermedius*1 GATGAAGTGGTGGCGTTCAGTCTAGCGCTGCCGATGTCCATGCCAGGGACGCAGTACTCCTCCGGCTTTACCGCAGAA

. . . . . . . . . . . . S M . . . . . L . . . . . .

*S. pallidus*20 GATGAAGCGGTGGCGTTCAGTCTAGCGACGTCGATATCCATGGCAGGGACACCGTACCTCTCCGGCTTTACCGCAGAA

D/N . . . . . . . . . P . Y L . . . . . . T . . . . .

*A. fragilis*10 rATGAAGCGGTGGCGTTCAGTCTAGCGACGCCGATATACTTGGCAGGGACGCCGTACTCCACCGGCTTTACCGCAGAA

. . . . . . . . . . . . T F T . . . . . S/L . . . . .

*H. pulcherrimus*5 GATGAAGCGGTGGCGTTCAGTCTAGCGACGTCGACGTTCACAGCAGGGACGCCTTACTCCTyGGGCTTTACCGCAGAA

. . . . . . . . . S . . T F T . . S F . . . L . . .

*S. franciscanus*4 GATGAAGCGGTGGCGTTCAGTCTAGCGTCGTCGACGTTCACCGCGGGGACGTCGTTCTCCTCCGGCCTAACCGCAGAA

. . . . . . . . . S . . T F T . . S F . . . L . . .

*S. nudus*1 GATGAAGCGGTGGCGTTCAGTCTAGCGTCGTCGACGTTTACAGCGGGGACGTCATTCTCCTCCGGCCTAACCGCAGAA

. . . . . . N . . S . . T F T . . S F . . . L . . .

*P. depressus*5 GATGAAGCGGTGGCGTTCAATCTAGCGTCGTCGACGTTTATCGCTGGGACGTCGTTCTCCTCCGGCCTAACCGCAGAA

*......*...........*.......**.*...*********.*.....****.*.**.***...*.*.........

Figure S11. Alignment of Illumina data for a section of SPU_021274 (*Sp-PkdL*) in exon 1. Underlined sites were removed prior to tests for positive selection. Positively selected codons are italicized and shown in bold in the Illumina alignment. Two positively selected codons occur in this region of exon 1 without any gaps in the alignment. The amino acids correspond to positions 107–132 in SPU_021274.

**Reference**

C R Q L G Y D H V H Q A I I S T N Y V S R P V H L

*S. purpuratus* TGTCGCCAACTCGGCTACGACCACGTTCACCAAGCTATCATCTCCACTAACTACGTTTCCAGGCCCGTTCACCTT

**Illumina**

C R Q L G Y D H V H Q A I I ***S T*** N Y V ***S*** R P V H L

*S. purpuratus* TGTCGCCAACTCGGCTACGACCACGTTCACCAAGCTATCATCTCCACTAACTACGTTTCCAGGCCCGTTCACCTT

. . . . . . . . . R . . . . . . . . . T . . . . .

*S. droebachiensis*6 TGTCGCCAACTCGGCTACGACCACGTTCGCCAAGCTATCATCTCCACTAACTACGTTACCAGGCCCGTTCACCTT

. . . . . . . . . H/R . . . . . . . . . T . . V/A . .

*S. intermedius*1 TGTCGmCAACTCGGCTACGACCACGTTCrCCAAGCTATCATyTCCACTAACTACGTTACCAGGCCCrTTCACCTT

. . . . . . . Y . Q . . . . Y . . . . F . . . . .

*S. pallidus*20 TGTCGCCAACTCGGCTACGACTACGTTCAGCAAGCTATCATCTACACTAACTACGTTTTCAGGCCCGTTCACCTT

. . . . . . . . . Q/R . . . . . . . . . . . . . . .

*A. fragilis*10 TGyCGCCAACTCGGCTACGACCACGTTCrmCAAGCTATCATCTCCACTAACTACGTTTCCAGrCCCGTTCACCTT

. . . . . . . . . . . . . . . . . . . . . . . . .

*H. pulcherrimus*5 TGTCGCCAACTCGGCTACGACCACGTTCACCAAGCTATCATCTCCACTAACTACGTTTCCAGGCCCGTTCACCTC

. . . . . . . . . F . . S . Y S Q . . . . . F . .

*S. franciscanus*4 TGTCGCCAACTCGGCTACGACCACGTTTTCCAAGCTAGCATATACTCACAATACGTTTCCAGGCCCTTTCACCTT

. . . . . . . . . F K . S . Y S Q . I Y . . F . .

*S. nudus*1 TGTCGCCAACTCGGCTACGACCACGTTTTCAAAGCTAGCATCTACTCACAATACATTTACAGGCCCTTTCACCTT

. . . . . . . . . F . . R . P I Q . . . . . . . .

*P. depressus*5 TGTCGCCAACTCGGCTACGACCACGTCTTCCAAGCTCGCATCCCCATACAATACGTTTCCAGGCCCGTTCACCTT

..*..*...............*....*****.....**...***.****.*...*..**...*...*.......*

Figure S12. Alignment of Illumina data for a section of SPU_024408 (*Sp-Srcr204*) in exon 9. Underlined sites were removed prior to tests for positive selection. Positively selected codons are italicized and shown in bold in the Illumina alignment. Three positively selected codons occur in this region of exon 9 without any gaps in the alignment. Visual inspection of the double mutation in *A. fragilis*10 on the UCSC *S. purpuratus* genome browser identified the phased alleles as Q and R. The amino acids shown represent positions 741–765 of SPU_024408.

**Reference**

G P P T P G P Q G P K G S Q G F K G S I G I D G I K

*S. purpuratus* GGACCCCCTACACCAGGCCCACAGGGCCCCAAAGGTTCACAAGGGTTCAAGGGAAGCATTGGCATTGATGGTATCAAA

**Illumina**

G P P T P G P Q G P K G ***S*** Q G ***F*** K G S I G I D G I K

*S. purpuratus* GGACCCCCTACACCAGGCCCACAGGGCCCCAAAGGTTCACAAGGGTTCAAGGGAAGCATTGGCATTGATGGTATmAAA

. . . . . . . . . . . . . . . . . . G . . . . . . .

*S. droebachiensis*6 GGACCCCCTACACCTGGACCACAGGGCCCCAAAGGTTCTCAAGGGTTCAAGGGAGGCATTGGCATTGATGGTATAAAG

. . . . . . . . . . . . . K . . . . G . . . . . . .

*S. intermedius*1 GGACCCCCTACACCAGGCCCACAGGGCCCCAAAGGTTCAAAAGGGTTCAAGGGTGGCATTGGCATTGATGGTATAAAA

. . . . . . . . . . . . . K . . . . G . . . . . I/R .

*S. pallidus*20 GGACCCCCTACACCAGGCCCACAGGGCCCCAAAGGTTCAAAAGGGTTCAAGGGTGGCATTGGCATTGATGGTAkAAAA

. . . . . . . . . . . . . K . . . . G . . . . . . .

*A. fragilis*10 GGACCCCCTACACCAGGCCCACAGGGCCCCAAAGGTTCAAAAGGTTTCAAGGGAGGCATTGGCATTGATGGTATmAAG

. . . . . . . . . . . . A K . . . . G . . . A . . .

*H. pulcherrimus*5 GGACCACCTACACCAGGCCCACAGGGCCCTAAAGGTGCAAAAGGGTTCAAAGGTGGCATTGGCATTGCTGGTATAAAA

. . . . . . . . . . . . Q K . G . . V . . . G . . .

*S. franciscanus*4 GGACCACCTACACCGGGCCCACAGGGCCCCAAAGGTCAAAAAGGGGGCAAGGGTGTAATTGGTATTGGAGGTATAAAA

. . . . . . . . . . . . Q K . . . . E L . . G . . .

*S. nudus*1 GGACCACCTACACCGGGCCCACAGGGCCCCAAAGGTCAAAAAGGGTTCAAGGGTGAACTTGGTATAGGAGGTATAAAA

. . S P L . . . . . . . . K . G . . E . . . R . . .

*P. depressus*5 GGACCATCTCCACTGGGCCCACAGGGCCCCAAAGGTTCAAAAGGGGGCAAGGGTGAAATTGGTATTAGAGGTATAAAA

.....**..*...**..*...........*......****....***...*..*****....*...***....**..*

Figure S13. Alignment of Illumina data for a section of SPU_028613 (*Sp-Fcolf_2*) in exon 20. The underlined site was removed prior to testing for positive selection. Positively selected codons are italicized and shown in bold in the Illumina alignment. Two positively selected codons occur in this region of exon 20 without any gaps in the alignment. The region corresponds to amino acids 572-597 in SPU_028613.

**Reference**

Q S S G V K V P S M N L E H M G Y F G S T S L A C

*S. purpuratus* CAGTCCAGTGGTGTTAAGGTACCGTCAATGAATTTAGAGCATATGGGCTATTTTGGAAGCACATCCCTTGCCTGT

**Illumina**

Q S S G ***V*** K V P S M N L E H M G Y F G S T ***S*** L A C

*S. purpuratus* CAGTCCAGTGGTGTTAAGGTACCGTCAATGAATTTAGAGCATATGGGCTATTTTGGAAGCACATCCCTTGCCTGT

. . . . . . . . . . . . . . . . . . . . . . . . .

*S. droebachiensis*6 CAGTCCAGTGGTGTAAAGGTACCGTCAATGAATTTAGAGCATATGGGCTACTTTGGAAGCACATCCCTTGCCTGT

. . . . M . . . . . . . Q . . . . . . . . . . . .

*S. intermedius*1 CAGTCCAGTGGTATGAAGGTACCGTCAATGAATTTACAGCATATGGGCTATTTTGGAAGCACATCCCTTGCCTGT

. . . . M . . . . . . . Q . . . . . . . T/A . . . .

*S. pallidus*20 CAGTCCAGTGGTATGAAGGTACCrTCAATGAATTTACAGCATATGGGCTATTTTGGAAGCrCATCCCTTGCCTGT

. . . R . . A . . . . . Q . . . . . . R . . . . .

*A. fragilis*10 CAGTCCAGTCGTGTAAAGGCACCCTCAATGAATTTACAGCATATGGGCTATTTTGGAAGAACATCCCTTGCCTGT

. . . . M . . . . . . . Q R . . . . A G . N . T .

*H. pulcherrimus*5 CAGTCCAGTGGTATGAAGGTACCGTCAATGAATTTACAGCGTATGGGCTATTTTGCAGGAACAAATCTTACCTGT

. . . . I . . . P . . . Q . . . . S A R . A L/F . .

*S. franciscanus*4 CAGTCCAGTGGTATTAAGGTACCACCAATGAATTTACAGCATATGGGCTATTCTGCAAGAACAGCCyTTGCCTGT

. . . . I . . . A . . . Q . . . . S A R . T . . .

*S. nudus*1 CAGTCCAGTGGTATTAAGGTACCAGCAATGAATTTACAGCATATGGGCTATTCTGCAAGAACAACCCTTGCCTGT

. . . . I . . . P . . . Q . . . . S A R . A . . .

*P. depressus*5 CAGTCCAGTGGTATTAAGGTACCACCAATGAATTTACAGCATATGGGCTATTCTGCAAGAACAGCCCTTGCCTGT

.........*..*.*....*...**...........*...*.........*.*..*.*.**..****..*.....

Figure S14. Alignment of Illumina data for a section of SPU_000649 (*Sp-Tnfaip3*) in exon 4. The underlined sites were removed prior to testing for positive selection. Positively selected codons are italicized and shown in bold in the Illumina alignment. Two positively selected codons occur in this region without any gaps in the alignment. The region shown corresponds to amino acids 451–475 in SPU_00649.

**Reference**

Q S R T A F C A T S D G T S E S V E I C

*S. purpuratus* CAGAGCCGTACTGCTTTCTGCGCCACATCTGATGGTACCTCTGAGTCAGTTGAGATCTGT

**Illumina**

Q S R T A F C A T S D G T S E S V E I C

*S. purpuratus* CAGAGCCGTACTGCTTTCTGCGCCACATCTGATGGTACCTCTGAGTCAGTTGAGATCTGT

E . . N . . . . . . E . V . . . . . . .

*S. droebachiensis*6 GAGAGCCGTAATGCTTTCTGCGCCACATCTGAGGGTGTCTCGGAGTCCGTTGAGATCTGT

. . . . . . . . . A/SE/T . A/T . . . . . . .

*S. intermedius*1 CAGAGCCGTACTGCTTTCTGyGCCACAkCTrmGGGTrCCTCkGAGTCmGTTGAGATCTGT

. . . . . . . . . . A . . . . . . . . .

*S. pallidus*20 CAGAGCCGTACTGCTTTCTGTGCCACATCTGCGGGTACCTCsGAGTCCGTTGAGATCTGT

. . . . . . . . . . . . . . . . . . . .

*A. fragilis10* CAGAGCCGTACTGCTTTCTGCGCCACATCTGATGGTACCTCTGAGTCAGTTGAGATCTGT

E . . N . . . T . . E . V . . . . . . .

*H. pulcherrimus*5 GAGAGTCGTAATGCTTTCTGTACCACATCTGAGGGTGTCTCTGAGTCAGTTGAGATCTGT

E . . N . . . . . . A . V . . . . . . .

*S. franciscanus*4 GAGAGCCGTAATGCTTTCTGTGCCACATCTGCGGGTGTCTCTGAGTCAGTCGAGATCTGT

E . . N . . . . . . A . V . . . . . . .

*S. nudus*1 GAGAGCCGTAATGCTTTCTGTGCCACATCTGCGGGTGTCTCTGAGTCAGTCGAGATCTGT

E . . . . . . . R . G . V . . . . . . .

*P. depressus*5 GAGAGCCGTACTGCTTTCTGTGCCAGATCTGGGGGTGTCTCTGAGTCAGTTGAGATCTGT

*....*....*.........**...*.*..***...**...*.....*..*.........

**Sanger**

E S R N A F C A T S E G V S E S V E I C

*S. droebachiensis*2 GAGAGCCGTAATGCTTTCTGCGCCACATCTGAGGGTGTCTCGGAGTCCGTTGAGATCTGT

E . . N . . . . . . E . . . . . . . . .

*S. droebachiensis*3 GAGAGCCGTAATGCTTTCTGCGCCACATCTGAGGGTGTCTCGGAGTCCGTTGAGATCTGT

E . . N . . . . . . E . . . . . . . . .

*S. droebachiensis*21 GAGAGCCGTAATGCTTTCTGCGCCACATCTGAGGGTGTCTCGGAGTCCGTTGAGATCTGT

Q . . T . . . . . A/SE/T . A/T . . . . . . .

*S. intermedius1* CAGAGCCGTACTGCTTTCTGyGCCACAkCTrmGGGTrCCTCkGAGTCmGTTGAGATCTGT

Q . . T . . . . . A E . A . . . . . . .

*S. intermedius*3 CAGAGCCGTACTGCTTTCTGTGCCACAGCTGAGGGTGCCTCGGAGTCAGTTGAGATCTGT

Q . . T . . . . . A E . A . . . . . . .

*S. intermedius5* CAGAGCCGTACTGCTTTCTGTGCCACAGCTGAGGGTGCCTCGGAGTCAGTTGAGATCTGT

Q . . T . . . . . . E . T . . . . . . .

*S. pallidus*1 CAGAGCCGTACTGCTTTCTGTGCCACATCTGCGGGTACCTCyGAGTCCGTTGAGATCTGT

Q . . T . . . . . . E . T . . . . . . .

*S. pallidus*2 CAGAGCCGTACTGCTTTCTGTGCCACATCTGCGGGTACCTCGGAGTCCGTCGAGATCTGT

Q . . T . . . . . . E . T . . . . . . .

*S. pallidus*4 CAGAGCCGTACTGCTTTCTGyGCCACATCTGCGGGTACCTCsGAGTCCGTTGAGATCTGT

Q . . T . . . . . . D . T . . . . . . .

*A. fragilis*2 CAGAGCCGTACTGCTTTCTGCGCCACATCTGATGGTACCTCTGAGTCAGTTGAGATCTGT

Q . . T . . . . . . D . T . . . . . . .

*A. fragilis*3 CAGAGCCGTACTGCTTTCTGCGCCACATCTGATGGTACCTCTGAGTCAGTTGAGATCTGT

Q . . T . . . . . . D . T . . . . . . .

*A. fragilis*4 CAGAGCCGTACTGCTTTCTGCGCCACATCTGATGGTACCTCTGAGTCAGTTGAGATCTGT

*.........*.........*......*..***...**...*.....*..*.........

Figure S15. Comparison of Illumina and Sanger alignment data for a region in exon 14 of SPU_000526 (*Sp-Ebr1*). Underlined sites were removed prior to testing for positive selection. *S. intermedius1* had 7 heterozygous mutations over a 28 bp region that resulted in 3 amino acid changes. Phasing identified the alleles as A-E-A and S-T-T. Sanger data from *S. intermedius*1 across exons 14 and 15 (369 bp) produced sequence identical to the Illumina results.

**Reference**

G G N F E P V V G S L C N P L L E P P S

*S. purpuratus* GGTGGTAATTTTGAGCCTGTCGTAGGTTCTCTCTGTAATCCTTTGTTAGAACCACCCAGT

**Illumina**

G G N F E P V V G S L C N P ***L*** L E P P S

*S. purpuratus* GGTGGTAATTTTGAGCCTGTCGTAGGTTCTCTCTGTAATCCTTTGTTAGAACCACCCAGT

. . . . . . . E . . . . . . A . . . . .

*S. droebachiensis*6 GGTGGTAATTTTGAGCCTGTTGAAGGATCTCTCTGTAACCCTGCCTTAGAACCACCCAGC

. . . . . . . E . . . . . . M . . . . .

*S. intermedius*1 GGTGGTAACTTTGAGCCTGTCGAAGGTTCTCTCTGCAACCCTATGTTAGAACCACCCAGT

. . . . . . . E . . . . . . M . . . . .

*S. pallidus*20 GGTGGTAACTTTGAGCCTGTTGAAGGTTCTCTCTGCAACCCTATGTTAGAACCACCCAGT

. . . . . . . A . . . . D . M . . . . .

*A. fragilis*10 GGTGGTAACTTTGAGCCTGTTGCAGGTTCTCTCTGTGACCCTATGTTAGAACCACCCAGT

. . . . Q . . E . . . . . . A . . . . .

*H. pulcherrimus*5 GGTGGCAGCTTCCAGCCTGTGGAAGGTTCTCTCTGCAACCCTGCGTTAGAACCACCCAGT

S . S . Q . . E . . . . . . A . . . . .

*S. franciscanus*4 AGTGGCAGCTTCCAGCCTGTCGAAGGTTCTCTCTGTAACCCTGCATTAGAACCACCCAGT

. . S . Q . . E . . . . . . A . . . . .

*S. nudus*1 GGTGGCAGCTTCCAGCCTGTCGAAGGTTCTCTCTGTAACCCTGCGTTAGAACCACCCAGT

. . S . Q . . D . . . . . . L . . . . .

*P. depressus*5 GGTGGCAGCTTCCAGCCTGTCGACGGTTCTCTCTGTAACCCTTTGTTAGAACCACCCAGT

*....*.**..**.......*.**..*........**.*...***..............*

**Sanger**

G G N F E P V V G S L C N P L L E P P S

*S. purpuratus*5 GGTGGTAATTTTGAGCCTGTCGTAGGTTCTCTCTGTAATCCTTTGTTAGAACCACCCAGT

. . . . . . . . . . . . . . . . . . . .

*S. purpuratus*7 GGTGGTAATTTTGAGCCTGTCGTAGGTTCTCTCTGTAATCCTTTGTTAGAACCACCCAGT

. . . . . . . E . . . . . . A . . . . .

*S. droebachiensis*5 GGTGGTAATTTTGAGCCTGTTGAAGGATCTCTCTGTAACCCTGCCTTAGAACCACCCAGC

. . . . . . . E . . . . . . A . . . . .

*S. droebachiensis*6 GGTGGTAATTTTGAGCCTGTTGAAGGATCTCTCTGTAACCCTGCCTTAGAACCACCCAGC

. . . . . . . E . . . . . . M . . . . .

*S. pallidus*1 GGTGGTAACTTTGAGCCTGTTGAAGGTTCTCTCTGCAACCCTATGTTAGAACCACCCAGT

. . . . . . . E . . . . . . M . . . . .

*S. pallidus*2 GGTGGTAACTTTGAGCCTGTTGAAGGTTCTCTCTGCAACCCTATGTTAGAACCACCCAGT

. . . . . . . A . . . . D . M . . . . .

*A. fragilis*2 GGTGGTAACTTTGAGCCTGTTGCAGGTTCTCTCTGTGACCCTATGTTAGAACCACCCAGT

S . S . Q . . E . . . . . . A . . . . .

*S. franciscanus*5 AGTGGCAGCTTCCAGCCTGTCGAAGGTTCTCTCTGTAACCCTGCATTAGAACCACCCAGT

S . S . Q . . E . . . . . . A . . . . .

*S. franciscanus*17 AGTGGCAGCTTCCAGCCTGTCGAAGGTTCTCTCTGTAACCCTGCATTAGAACCACCCAGT

. . S . Q . . E . . . . . . A . . . . .

*S. nudus*2 GGTGGCAGCTTCCAGCCTGTCGAAGGTTCTCTCTGTAACCCTGCGTTAGAACCACCCAGT

*....*.**..**.......*.*...*........**.*...***..............*

Figure S16. Comparison of Illumina and Sanger alignment data for a region of exon 21 in SPU_000526 (*Sp-Ebr1*). Positively selected codons are bolded and italicized in the Illumina alignment. A single allele was cloned from each individual for the Sanger data as described in Pujolar and Pogson (2011). Identical Sanger and Illumina sequences were obtained for *S. droebachiensis*6 over 168 bp of exon 21. The region shown corresponds to amino acids 651–670 of SPU_000526.

**Reference**

S E N V S L G H H V I T V V A E D M D L

*S. purpuratus* TCTGAGAACGTATCCCTCGGCCATCATGTCATCACCGTCGTCGCGGAGGACATGGATCTG

**Illumina**

S E N V S L G H H V I T V V A E D M D L

*S. purpuratus* TCTGAGAACGTATCCCTCGGCCATCATGTCATCACCGTCGTCGCGGAGGACATGGATCTG

. . . . . . . . L/H . . . . . . . . . . .

*S. droebachiensis*6 TCwGAGAACGTATCmCTyGGCCAyCwTGTCATCACCGTCGTCGCGGArGACATGGATCTG

. . . . . . . . L . . . . . . . . . . .

*S. intermedius*1 TCTGAGAATGTATCCCTCGGCCATCTTGTCATCACCGTTGTCGCGGAGGACATGGATCTG

. . . . . . . . . . . . . . . . . . . .

*S. pallidus*20 TCCGAGAACGTATCCCTyGGCCATCATGTCATCACCGTCGTCGCGGAGGACATGGATCTG

. . . . . . . . . . . . . . . . . . . .

*A. fragilis*10 TCTGAGAACGTATCCCTCGGCCATCATGTCATCACCGTTGTCGCrGAGGACATGGATCTG

. . . . . . . . . . . . . . . . . . . .

*H. pulcherrimus*5 TCTGAGAACGTATCCCTCGGCCATCATGTCATCACCGTCGTCGCGGAGGACATGGATCTG

. . . . . . . . . . . . . . . . . . . .

*S. franciscanus*4 TCTGAGAACGTATCCCTCGGCCATCATGTCATCACCGTAGTCGCGGAGGACATGGATCTG

. . . . . . . . . . . . . . . . . . . .

*S. nudus*1 TCTGAGAACGTATCCCTCGGCCACCATGTCATCACCGTAGTCGCGGAGGACATGGATCTG

. . . . . . . . . . . . . . . . . . . .

*P. depressus*5 TCTGAGAACGTATCCCTCGGCCATCATGTCATCACsGTAGTCGCGGAGGACATGGATCTG

..*...........*..*.....*.*.........*........*..*............

**Sanger**

S E N V S L G H L/H V I T V V A E D M D L

*S. droebachiensis*3 TCTGAGAAyGTATCCCTCGGCCAyCwTGTCATCACCGTyGTCGCGGArGACATGGAyCTG

. . . . . . . . H . . . . . . . . . . .

*S. droebachiensis*4 TCTGAGAACGTATCCCTCGGCCACCATGTCATCACCGTCGTCGCGGAAGACATGGAyCTG

. . . . . . . . H . . . . . . . . . . .

*S. droebachiensis*5 TCTGAGAACGTATCCCTCGGCCACCATGTCATCACCGTCGTCGCGGAAGACATGGACCTG

. . . . . . . . L/H . . . . . . . . . . .

*S. droebachiensis*6 TCwGAGAACGTATCmCTyGGCCAyCwTGTCATCACCGTCGTCGCGGArGACATGGATCTG

. . . . . . . . H . . . . . . . . . . .

*S. pallidus*1 TCCGAGAACGTATCCCTTGGCCATCATGTCATCACCGTCGTCGCGGAGGACATGGATCTG

. . . . . . . . H . . . . . . . . . . .

*S. pallidus*2 TCTGAGAACGTATCCCTCGGCCATCATGTyATCACCGTyGTCGCGGAGGACATGGATCTG

. . . . . . . . L/H . . . . . . . . . . .

*S. pallidus*4 TCCGAGAACGTATCCCTyGGCCAyCwTGTCATCACCGTyGTCGCGGAGGACATGGATCTG

. . . . . . . . H . . . . . . . . . . .

*S. pallidus*7 TCTGAGAACGTATCCCTyGGCCATCAyGTCATCACCGTyGTCGCGGAGGACATGGATCTG

..*.....*.....*..*.....*.**...........*........*........*...

Figure S17. Comparison of Illumina and Sanger alignment data for a region in exon 19 of SPU_001452 (*Sp-Dachs*). Amino acids removed prior to PAML tests are underlined in the Illumina alignment. Sanger sequencing of *S. droebachiensis*6 produced identical sequence for 699 bp of the Illumina-generated alignment (not shown). The protein region presented corresponds to amino acids 2446–2465 of SPU_001452.

**Reference**

G T C F D G I N G Y D C E C V E G F G G

*S. purpuratus* GGAACATGCTTTGATGGAATCAATGGCTACGACTGCGAATGTGTCGAGGGTTTTGGTGGT

**Illumina**

G T C F D G I N G Y D C E C V E G F G G

*S. purpuratus* GGAACATGCTTTGATGGAATCAATGGmTACGACTGCGAATGTGTCGAGGGTTTTGGTGGT

. . . L/F . . . . D/G . . . . . . D . . S/G .

*S. droebachiensis*6 GGAACATGyTTkGATGGAATCAATGrTTACGACTGCGAATGyGTCGATGGyTTTrGTGGy

. . . . . . . . . . . . . . . D . . . .

*S. intermedius*1 GGAACATGCTTTGATGGAATCAATGGTTACGACTGCGAATGTGTCGATGGTTTTGGTGGT

. . . . . . . D/N . . . . . . . D . . . .

*S. pallidus*20 GGAACATGCTTTGATGGAATCrATGGTTACGACTGCGAATGTGTCGATGGCTTTGGTGGG

. . . . . . . . . . E/D . . . . H/D . . . .

*A. fragilis*10 GGAACATGCTTTGATGGAATmAATGGTTACGAsTGCGArTGTGTCsATGGyTTTGGTGGT

. . . . . . . . . . . . . . . D . . . .

*H. pulcherrimus*5 GGAACATGCTTTGATGGAATCAATGGTTACGACTGCGAATGTGTCGATGGCTTTGGTGGT

. . . . . . . . . . . . . . . D . . . .

*S. franciscanus*4 GGAACATGTTTTGATGGAATCAATGGTTATGACTGCGAATGTGTCGATGGTTTTGGCGGT

. . . . . . . . . . . . . . . V/D . . . .

*S. nudus*1 GGGACATGTTTTGATGGAATCAATGGTTATGACTGCGAATGTGTCGwTGGTTTTGGCGGT

. . . . . . . . . . . . . . . G . . . .

*P. depressus*5 GGAACGTGTTTTGATGGAATCAATGGTTATGACTGCGAATGTGTCGGTGGTTTTGGCGGT

..*..*..*..*........**...**..*..*.....*..*...***..*...*.*..*

**Sanger**

G T C F D R/G I N G Y D C E C V D G F G G

*S. droebachiensis*1 GGAACATGCTTTGATrGAATCAATGGTTACGACTGCGAATGTGTCGATGGTTTTGGTGGT

. . . . . G . . . . . . . . . D . . . .

*S. droebachiensis*5 GGAACATGCTTTGATGGAATCAATGGTTACGACTGCGAATGTGTCGATGGTTTTGGTGGT

. . . . . G . . D/G . . . . . . D . . . .

*S. droebachiensis*7 GGAACATGyTTTGATGGAATCAATGrTTACGACTGCGAATGyGTCGATGGTTTTrGTGGT

. . . L . G . . . . . . . . . D . . . .

*S. droebachiensis*9 GGAACATGCTTGGATGGAATCAATGGTTACGACTGCGAATGTGTCGATGGCTTTGGTGGC

. . . . . G . D/N . . . . . . . D . . . .

*S. pallidus*1 GGAACATGyTTTGATGGAATCrATGGTTACGACTGCGAATGyGTCGATGGyTTTGGTGGk

. . . . . G . D/N . . . . . . . D . . . .

*S. pallidus*4 GGAACATGyTTTGATGGAATCrATGGTTACGACTGCGAATGyGTCGATGGyTTTGGTGGk

. . . . . G . . . . . . E/Q . . H/D . . . .

*S. pallidus*7 GGAACmTGCTTTGATGGAATCAATGGTTACGACTGCsAATGTGTCsATGGTTTTGGTGGT

. . . . . G . D/ND/G . . . . . . D . . . .

*S. pallidus*10 GGAACmTGCTTTGATGGAATCrATGrTTACGACTGCGAATGTGTCGATGGCTTTGGTGGk

.....*..*..*...*.....*...*..........*....*...*....*...*....*

Figure S18. Comparison of Illumina and Sanger alignment data for a region in exon 7 of SPU_005955 (*Sp-NotchL*). Amino acids removed prior to PAML tests are underlined. Sanger data was obtained by directly sequencing PCR products. The region shown represents amino acids 2672–2691 of SPU_005955.

**Reference**

Y V D Q V L L R D K N L D N L L A V E E

*S. purpuratus* TACGTGGACCAAGTTTTATTGCGAGATAAAAATCTGGACAATTTACTAGCAGTGGAAGAG

**Illumina**

Y V D Q V L L R D K N L D N L L A V E E

*S. purpuratus* TACGTGGACCAAGTTTTATTGCGAGATAAAAATCTGGACAATTTACTAGCAGTGGAAGAG

. . . . . . . . . . . . . . . . . . . .

*S. droebachiensis*6 TACGTGGACCAAGTTTTATTGCGAGATAAAAATCTGGACAATTTACTAGCCGTGGAAGAG

. . . Q/K . . . . . . . . . . . . . . . .

*S. intermedius*1 TACGTGGACmAAGTTTTATTGCGAGATAAAAATCTGGACAATyTACTAGCmGTGGAAGAG

. . . Q/K . . . . . . . . . . . . . . . .

*S. pallidus*20 TACGTGGACmAAGTTTTATTGCGAGATAAAAATCTGGACAATyTACTAGCCGTGGAAGAG

. . . . . . . . . . . . . . . . . . . .

*A. fragilis*10 TACGTGGACCAAGTTTTATTGCGAGATAAAAATCTGGACAATTTACTAGCAGTGGAAGAG

. . . . . . . . . . . . . . . . . . . .

*H. pulcherrimus*5 TACGTGGACCAAGTTTTATTGCGAGATAAAAATCTGGACAATTTACTAGCAGTGGAAGAG

. . . . . . . . . . . . . . . . . . . .

*S. franciscanus*4 TACGTGGACCAAGTCTTATTGCGAGATAAAAATCTGGACAATTTACTAGCGGTGGAAGAG

. . . . . . . . . . . . . . . . . . . .

*S. nudus*1 TACGTGGACCAAGTCTTATTGCGAGATAAAAATCTGGACAATTTATTAGCGGTGGAAGAG

. . . . . . . . N . . . . . . . . . . .

*P. depressus*5 TACGTGGACCAAGTCTTATTGCGAAATAAAAATTTGGACAATTTATTAGCCGTGGAAGAG

.........*....*.........*........*........*..*....*.........

**Sanger**

Y V D Q V L L R D K N L D N L L A V E E

*S. purpuratus*1 TACGTGGACCAAGTTTTATTGCGAGATAAAAATCTGGACAATTTACTAGCAGTGGAAGAG

. . . . . . . . . . . . . . . . . . . .

*S. purpuratus*2 TACGTGGACCAAGTTTTATTGCGAGATAAAAATCTGGACAATTTACTAGCAGTGGAAGAG

. . . . . . . . . . . . . . . . . . . .

*S. droebachiensis*1 TACGTGGACCAAGTTTTATTGCGAGATAAAAATCTGGACAATTTACTAGCCGTGGAAGAG

. . . . . . . . . . . . . . . . . . . .

*S. droebachiensis*2 TACGTGGACCAAGTTTTATTGCGAGATAAAAATCTGGACAATTTACTAGCAGTGGAAGAG

. . . . . . . . . . . . . . . . . . . .

*S. pallidus*1 TACGTGGACCAAGTTTTATTGCGAGATAAAAATCTGGACAATTTACTAGCCGTGGAAGAG

. . . . . . . . . . . . . . . . . . . .

*S. pallidus*2 TACGTGGACCAAGTTTTATTGCGAGATAAAAATCTGGACAATTTACTAGCCGTGGAAGAG

. . . . . . . . . . . . . . . . . . . .

*A. fragilis*2 TACGTGGACCAAGTTTTATTGCGAGATAAAAATCTGGACAATTTACTAGCAGTGGAAGAG

. . . . . . . . . . . . . . . . . . . .

*A. fragilis*3 TACGTGGACCAAGTTTTATTGCGAGATAAAAATCTGGACAATTTACTAGCAGTGGAAGAG

. . . . . . . . . . . . . . . . . . . G

*S. franciscanus*1 TACGTGGACCAAGTCTTATTGCGAGATAAAAATCTGGACAATTTACTAGCGGTGGAAGGG

. . . . . . . . . . . . . . . . . . . .

*S. nudus*1 TACGTGGACCAAGTCTTATTGCGAGATAAAAATCTGGACAATTTATTAGCGGTGGAAGAG

. . . . . . . . . . . . . . . . . . . .

*S. nudus*2 TACGTGGACCAAGTCTTATTGCGAGATAAAAATCTGGACAATTTATTAGCGGTGGAAGAG

..............*..............................*....*.........

Figure S19. Comparison of alignment data for SPU_007013 in exon 1 of cyclinD (*Sp-CycD*). Underlined codons in the Illumina alignment were removed prior to testing for positive selection. Sanger sequences were obtained by cloning one allele per individual as described in Addison and Pogson (2009). Identical Illumina and Sanger sequences were obtained for *S. nudus*1 for 213 bp of exon 1 (not shown). The region presented corresponds to amino acids 21–40 of SPU_007013.

**Reference**

A E Y V V F F Q D Y T R K Q R F N N A T

*S. purpuratus* GCGGAATACGTGGTTTTTTTCCAAGATTATACGCGCAAACAGCGTTTCAATAACGCAACT

**Illumina**

***A*** E Y ***V V*** F F ***Q*** D Y ***T R*** K ***Q*** R F N N ***A*** T

*S. purpuratus* GCGGAATACGTGGTTTTTTTCCAAGATTATACGCGCAAACAGCGTTTCAATAACGCAACT

L . . K E . . E . . I N . S . L D . . .

*S. droebachiensis*6 TTGGAATACAAGGAATTTTTCGAAGACTATATTAATAAGAGCCGTTTGGATAACGCAACT

L . . S D . . K . . I D . . . . . . . .

*S. intermedius*1 TTGGAATACTCGGATTTTTTCAAAGATTATATAGACAAACAGCGTTTCAATAACGCAACT

L . . K E . . E . . I N . S . L D . . .

*S. pallidus*20 TTGGAATACAAGGAATTTTTCGAAGACTATATTAATAAGAGCCGTTTGGATAACGCAACT

L . . K E . . . . . K N . . -.----- . . .

*A. fragilis*10 TTGGAATACAAGGAATTTTTCCAAGACTATAAGAACAAACAGNGNNNNNATAACGCAACT

. . . L E . . D . . K N . . . . . . . .

*H. pulcherrimus*5 GCGGAATAyCTGGAATTTTTCGATGATTATAAGAACAAACAGCGTTTCAATAACGCAACT

. . . T K Y . E E . I S . . H D T . V S

*S. franciscanus*4 GCGGAATACACGAAATATTTCGAAGAATATATTAGCAAACAGCATGACACGAACGTATCT

. . F Q E . . R A . K Q . . H . . . V S

*S. nudus*1 GCGGAATTCCAGGAATTTTTCAGAGCTTATAAGCAAAAACAGCATTTCAATAACGTATCT

E . F Q K Y . A E . . H . . . D T . G S

*P. depressus*5 GAGGAATTCCAGAAATATTTTGCAGAATATACACACAAACAGCGTGACACGAACGGATCT

**.....****.***.*...****.**....*****..*************....*.*..

**Sanger**

L E Y K D F F E D Y I N K S R L D N A T

*S. droebachiensis*27 TTGGAATACAAGGATTTTTTCGAAGACTATATTAATAAGAGCCGTTTGGATAACGCAACT

. . . . E . . . . . . . . . . . . . . .

*S. droebachiensis*28 TTGGAATACAAGGAATTTTTCGAAGACTATATTAATAAGAGCCGTTTGGATAACGCAACT

. . . . E . . . . . . . . . . . . . . .

*S. droebachiensis*35 TTGGAATACAAGGAATTTTTCGAAGACTATATTAATAAGAGCCGTTTGGATAACGCAACT

. . . S . . . K . . . D . Q . F N . . .

*S. intermedius*1 TTGGAATACTCGGATTTTTTCAAAGATTATATAGACAAACAGCGTTTCAATAACGCAACT

. . . S . . . K . . . D . Q . F N . . .

*S. intermedius*3 TTGGAATACTCGGATTTTTTCAAAGATTATATAGACAAACAGCGTTTCAATAACGCAACT

. . . S . . . K . . . D . Q . F N . . .

*S. intermedius*6 TTGGAATACTCGGATTTTTTCAAAGATTATATAGACAAACAGCGTTTCAATAACGCAACT

. . . . E . . . . . . . . . . . . . . .

*S. pallidus*2 TTGGAATACAAGGAATTTTTCGAAGACTATATTAATAAGAGCCGTTTGGATAACGCAACT

. . . . E . . . . . . . . . . . . . . .

*S. pallidus*4 TTGGAATACAAGGAATTTTTCGAAGACTATATTAATAAGAGCCGTTTGGATAACGCAACT

. . . . E . . . . . . . . . . . . . . .

*S. pallidus*42 TTGGAATACAAGGAATTTTTCGAAGACTATATTAATAAGAGCCGTTTGGATAACGCAACT

. . . . E . . Q . . K . . Q . . . . . .

*A. fragilis*2 TTGGAATACAAGGAATTTTTCCAAGACTATAAGAACAAACAG------GATAACGCAACT

. . . . E . . Q . . K . . Q . . . . . .

*A. fragilis*3 TTGGAATACAAGGAATTTTTCCAAGACTATAAGAACAAACAG------GATAACGCAACT

. . . . E . . Q . . K . . Q . . . . . .

*A. fragilis*4 TTGGAATACAAGGAATTTTTCCAAGACTATAAGAACAAACAG------GATAACGCAACT

.....*...**...*......*....*....**..*..***********...........

Figure S20. Comparison of Illumina and Sanger alignment data for a region in exon 1 of SPU_008159 (*Sp-Kcnk13*). Positively selected codons are bolded and italicized in the Illumina alignment. Amino acids removed prior to PAML tests are underlined. The two missing amino acids in *A. fragilis* resulted in the filtering of three codons from the Illumina data. The region presented corresponds to amino acids 51–70 of SPU_008159.

**Reference**

K A L Q K S N S L E T S P D T T F P E F

*S. purpuratus* AAAGCACTCCAAAAGAGTAACAGTCTTGAAACATCCCCTGACACTACTTTCCCAGAATTT

**Illumina**

K A L Q K S N S L E T P/S P D T T F P E F

*S. purpuratus* AAAGCACTCCAAAAGAGTAACAGTCTTGAAACAyCCCCTGACACTACTTTCCCAGAATTT

. . I/L . . . . . . . . S . . . S . Q . C/S

*S. droebachiensis*6 AAAGCAmTCCAAAAGAGyAACAGTCTTGAAACATCyCCyGACACTwsTTTCCAAGAAwGT

. . I . . . . . . K . S . . . . C . . .

*S. intermedius*1 AAAGCACTCCAAAAGAGTAACAGTCTTAAAACATCTCCTGACACTACTTGCCCAGAATTT

. . I/L . . . . . . . . S . . . S/TF/CQ/P . C/F

*S. pallidus*20 AAAGCAmTCCAAAAGAGTAACAGTCTTGAAACATCCCCTGACACTAsTTkCCmAGAATkT

. . . . . . . . . . . S . . . . Y/F . . .

*A. fragilis*10 AAAGCACTyCAACAGAGTAACAGTCTTGAAACATCCCCTGACACTACTTwCCCAGAATTT

. V/A . . . . . . . . . S . . I/TS/T . . . .

*H. pulcherrimus*5 AAAGyACTCCAAAAGAGTAACAGTCTTGAAACATCCTCTGACAyTwCTTTCCAAGAATTT

. . . . . . . . . . . S . . . . . . . .

*S. franciscanus*4 AAAGCACTCCAAAAGAGTAACAGTCTTGAAACATCCCCTGACACTACTTTCCCAGAATTT

. . . . . . . . . . . S . . . . . . . .

*S. nudus*1 AAAGCACTCCAAAAGAGTAACAGTCTTGAATCATCCCCTGACACTACTTTCCCAGATTTT

. . . . . . . . . . . S . . . . . . . .

*P. depressus*5 AAAGCACTCCAAAAGAGTAACAGTCTTGAAACATCCCCTGATACTACTTTTCCAGAATTT

....*.*.*...*....*..........*.*..*.**.*..*.*.**..**.*...***.

**Sanger**

K A I/L Q K S N S L E T S P D T S/TC/FQ/P E C/F

*S. droebachiensis*4 AAAGCAmTCCAAAAGAGTAACAGTCTTGAAACATCCCCTGACACTAsTTkCCmAGAATkT

. . I . . . . . . . . . . . . S F Q . C

*S. droebachiensis*5 AAAGCAATCCAAAAGAGTAACAGTCTTGAAACATCCCCTGACACTAGTTTCCAAGAATGT

. . I/L . . . . . . . . . . . . S F Q . C/S

*S. droebachiensis*6 AAAGCAmTCCAAAAGAGyAACAGTCTTGAAACATCyCCyGACACTwsTTTCCAAGAAwGT

. . I/L . . . . . . . . . . . . S/T F C/Q . C/F

*S. droebachiensis*7 AAAGCAmTCCAAAAGAGTAACAGTCTTGAAACATCCCCTGACACTAsTTTCCmAGAwTkT

. . L . . . . . . . . A/S . . . T C P . F

*S. pallidus*1 AAAGCACTCCAAAAGAGTAACAGTCTTGAAACAkCCCCTGACACTACTTGCCCAGAATTT

. . L . . R/S . . . . . . . . . S/TF/CQ/P . S/F

*S. pallidus*2 AAAGCACTCCAAAAGAGwAACAGTCTTGAAACATCyCCyGACACTwCTTkCCmAGAAwkT

. . L . . . . . . . . . . . . T C P . F

*S. pallidus*4 AAAGCACTCCAAAAGAGTAACAGTCTTGAAACATCCCCTGACACTACTTGCCCAGAATTT

. . L . . . . . . . . . . . . T F Q . C

*S. pallidus*7 AAAGCACTTCAAAAGAGTAACAGTCTkGAAACATCCCCTGACACTACTTTCCAAGAATGT

......*.*........*........*......*.*..*......**..*..*...***.

Figure S21. Comparison of Illumina and Sanger alignment data for a region in exon 17 of SPU_009691 (*Sp-Plceta*). Amino acids removed prior to PAML tests are underlined in the Illumina alignment. *S. droebachiensis*6 was sequenced by both methods and produced identical sequence across 771 bp (not shown). The double heterozygous mutations at amino acid 16 in *S. droebachiensis*6 both code for serine (inferred from the phased data). The region shown corresponds to amino acids 2283–2302 of SPU_009691.

**Reference**

I K G L Q C P V C E K T F T R R S G V S

*S. purpuratus* ATAAAAGGCTTGCAGTGTCCGGTGTGTGAGAAAACATTCACAAGACGCTCCGGTGTCAGC

**Illumina**

I K G L Q C P V C E K T F T R R S G V S

*S. purpuratus* ATAAAAGGCTTGCAGTGTCCGGTGTGTGAGAAAACATTCACrAGACGCTCCGGTGTCAGC

. . . . . . . . . . . . . . . . . . . .

*S. droebachiensis*6 ATAAAAGGyTTGCAGTGCCCGGTGTGTGAGAArACATTCACAAGACGCTCCGGTGTyAGC

. . . . . . . . . . . . . . . . . . . .

*S. intermedius*1 ATAAAAGGCTTGCAGTGCCCGGTGTGTGAGAAAACATTCACAAGACGyTCCGGTGTCAGC

. . . . . . . . . . . . . . . . . . . .

*S. pallidus*20 ATAAAAGGCTTGCAGTGCCCGGTGTGTGArAAAACATTCACGAGACGTTCCGGTGTCAGC

. . . . . . . . . . . . . . . . . . . .

*A. fragilis*10 ATCAAAGGCTTGCAGTGCCCGGTGTGTGAGAAAACATTCACAAGACGTTCCGGTGTCAGC

. . . . . . . . . . . . . . . . . . . .

*H. pulcherrimus*5 ATAAAAGGCTTGCAGTGCCCGGTGTGTGAGAAAACATTCACAAGACGTTCCGGTGTCAGC

. . . . . . . . . . . . . . . . . . . .

*S. franciscanus*4 ATAAAAGGCTTGCAGTGCCCAGTGTGTGAGAAAACATTCACAAGACGTTCTGGTGTCAGC

. . . . . . . . . . . . . . . . . . . .

*S. nudus*1 ATAAAAGGCCTGCAGTGCCCAGTGTGTGAGAAAACATTCACAAGACGTTCTGGTGTCAGC

. . . . . . . . . . . . . . . . . . . .

*P. depressus5* ATAAAAGGCTTGCAGTGCCCAGTGTGTGAGAAAACATTCACAAGACGTTCTGGTGTCAGC

..*.....**.......*..*........*..*........*.....*..*.....*...

**Sanger**

I K G L Q C P V C E K T F T R R S G V S

*S. droebachiensis*1 ATAAAAGGCTTGCAGTGCCCGGTGTGTGAGAAAACATTCACAAGACGCTCCGGTGTTAGC

. . . . . . . . . . . . . . . . . . . .

*S. droebachiensis*4 ATAAAAGGCTTGCAGTGCCCGGTGTGTGAGAAAACATTCACAAGACGCTCCGGTGTTAGC

. . . . . . . . . . . . . . . . . . . .

*S. droebachiensis*5 ATAAAAGGyTTGCAGTGCCCGGTGTGTGAGAArACATTCACAAGACGCTCCGGTGTyAGC

. . . . . . . . . . . . . . . . . . . .

*S. droebachiensis*7 ATAAAAGGyTTGCAGTGCCCGGTGTGTGAGAArACrTTCACAAGACGCTCCGGTGTyAGC

. . . . . . . . . . . . . . . . . . . .

*S. pallidus*4 ATAAAAGGCTTGCAGTGCCCGGTGTGTGAGAAAACATTCACAAGACGTTCCGGTGTCAGC

. . . . . . . . . . . . . . . . . . . .

*S. pallidus*7 ATAAAAGGCTTGCAGTGCCCGGTGTGTGAGAAAACATTCACmAGACGCTCyGGTGTCAGC

........*.......................*..*.....*.....*..*.....*...

Figure S22. Comparison of Illumina and Sanger alignment data for a region in exon 6 of SPU_012948 (*Sp-Z50*). Amino acids removed prior to PAML tests are underlined in the Illumina alignment. The region corresponds to amino acids 1447–1466 of SPU_012948.

**Reference**

H P A I R P T M P L P T R P T A A T A L

*S. purpuratus* CACCCAGCGATTCGGCCAACCATGCCCCTACCGACAAGACCGACAGCGGCGACGGCTCTC

**Illumina**

H P A I R P T M P L P T R P T A A T A L

*S. purpuratus* CACCCAGCGATTCGGCCAACCATGCCCCTACCGACAAGACCGACAGCGGCGACGGCTCTC

. . . . . . . . . . . . . . . . ------ . .

*S. droebachiensis*6 CACCCAGCGATTCGGCCAACCATGCCCCTGCCAACAAGACCGACGGCGGCNNCGGCTCTC

. . . . . . . . . . . . . . . . . . . .

*S. intermedius*1 CACCCAGCGATTCGCCCAACCATGCCCCTGCCAACrAGACCGACrGCGGCGACGGCTCTC

. . . . . . . . . . . . . . . . . --- . .

*S. pallidus*20 CACCCAGCGATTCGGCCAACCATGCCCCTACCAACAAGACCGACGGCGGCGNCGGCTCTC

. . . . . . . . . . . . . . . . . . . .

*A. fragilis*10 CACCCAGCGATTCGGCCAACCATGCCCCTGCCGACAAGACCGACAGCGGCGACGGCTCTC

. . . . . . . . . . . . . . . . ----- . .

*H. pulcherrimus*5 CACCCAGCGATTCGGCCAACCATGCCCCTGCCGACAAGACCGACAGCGGCNNCGGCTCTC

. . . . . . . . . . . . . . . . . . . .

*S. franciscanus*4 CACCCAGCGATTCGGCCAACCATGCCCCTGCCGACAAGACCGACAGCGGCGACGGCTCTC

. . . . . . . . . . . . . . . . . . . .

*S. nudus*1 CACCCAGCGATTCGGCCAACCATGCCCCTGCCGACAAGACCGACAGCGGCGACGGCTCTC

. . . . . . . . . . . . . . . . . . . .

*P. depressus*5 CACCCAGCGATTCGGCCAACCATGCCCCTGCCGACAAGACCGACAGCGGCGACGGCTCTC

..............*..............*..*..*........*.....**........

**Sanger**

H P A I R P T M P L P T R P T A A T A L

*S. purpuratus*1 CACCCAGCGATTCGGCCAACAATGCCCCTACCAACAAGACCGACAGCGGCGACGGCTCTC

. . . . . . . . . . . . . . . . . . . .

*S. purpuratus*5 CACCCAGCGATTCGGCCTACCATGCCCCTACCAACAAGACCGACAGCGGCGACGGCTCTC

. . . . . . . . . . . . . . . . ------ . .

*S. droebachiensis*5 CACCCAGCGATTCGGCCAACCATGCCCCTGCCAACAAGACCGACGGCG------GCTCTC

. . . . . . . . . . . . . . . . ------ . .

*S. droebachiensis*11 CACCCAGCGATTCGGCCAACCATGCCCCTGCCAACAAGACCGACGGCG------GCTCTC

. . . . . . . . . . . . . . . . ------ . .

*S. pallidus*1 CACCCAGCGATTCGGCCAACCATGCCCCTACCAACAAGACCGACGGCG------GCTCTC

. . . . . . . . . . . . . . . . ------ . .

*S. pallidus*2 CACCCAGCGATTCGGCCAACCATGCCCCTACCAACAAGACCGACGGCG------GCTCTC

. . . . . . . . . . . . . . . . . . . .

*A. fragilis*2 CACCCAGCGATTCGCCCAACCATGCCCCTGCCAACGAGACCGACAGCGGCGACGGCTCTC

. . . . . . . . . . . . . . . . . . . .

*A. fragilis*3 CACCCAGCGATTCGCCCAACCATGCCCCTGCCAACGAGACCGACAGCGGCGACGGCTCTC

. . . . . . . . . . . . . . . . . . . .

*S. franciscanus*1 CACCCAGCGATTCGGCCAACCATGCCCCTGCCGACAAGACCGACAGCGGCGACGGCTCTC

. . . . . . . . . . . . . . . . . . . .

*S. franciscanus*3 CACCCAGCGATTCGGCCAACCATGCCCCTGCCGACAAGACCGACAGCGGCGACGGCTCTC

. . . . . . . . . . . . . . . . . . . .

*S. nudus*1 CACCCAGCGATTCGGCCAACCATGCCCCTGCCGACAAGACCGACAGCGGCGACGGCTCTC

. . . . . . . . . . . . . . . . . . . .

*S. nudus*2 CACCCAGCGATTCGGCCAACCATGCCCCTGCCGACAAGACCGACAGCGGCGACGGCTCTC

..............*..*..*........*..*..*........*...******......

Figure S23. Comparison of Illumina and Sanger alignment data for a region in exon 1 of SPU_025133 (*Sp-SoxB2*). Amino acids removed prior to PAML tests are underlined in the Illumina alignment. The Sanger data was obtained by cloning as described in Addison and Pogson (2009). Identical sequences (810 bp) were obtained from *S. nudus1* for the Illumina and Sanger data (not shown). The region corresponds to amino acids 250–269 of SPU_02513.

**Reference**

G A Q G M G G P V G G G P M G G P P Q F

*S. purpuratus* GGCGCTCAAGGAATGGGTGGACCAGTCGGAGGTGGACCGATGGGTGGACCTCCACAATTT

**Illumina**

G A Q G M G G P V G G G/V P M G G P P Q F

*S. purpuratus* GGCGCTCAAGGAATGGGTGGACCAGTCGGAGGTGkACCGATGGGTGGACCTCCACAATTT

. . . . . . . . . . . G Q . . R . . . .

*S. droebachiensis*6 GGTGCTCAAGGAATGGGTGGACCAGTCGGTGGTGGACAGATGGGTAGACCTCCACAATTT

. . . . . . . . . . . --------- . . A . . .

*S. intermedius*1 GGCGCTCAAGGAATGGGTGGACCAGTCGGAGGTGNNNNGANGGGTGGAGCTCCACAATTT

. . . . . . R . . . . G . . . . . . . .

*S. pallidus*20 GGCGCTCAAGGAATGGGTAGACCAGTCGGAGGTGGACCGATGGGTGGACCTCCACAATTT

. . . . . . . . . . . G . . . . . . . .

*A. fragilis*10 GGCGCTCAAGGAATGGGTGGACCAGTCGGAGGTGGACCGATGGGTGGACCTCCGCAATTT

. . . . . . A . . . . G . . . . . . . .

*H. pulcherrimus*5 GGCGCTCAAGGAATGGGTGCACCAGTCGGAGGTGGACCGATGGGTGGrCCTCCACAATTT

. . . . . . . A F G/R . ---------------------------

*S. franciscanus*4 GGCGCCCAAGGGATGGGTGGAGCATTTrGAGGTGNNNNNNNNNNNNNNNNNNNNNNNNNN

. T . . . . . A . . . ---------------------------

*S. nudus*1 GGCACCCAAGGGATGGGTGGGGCAGTTGGAGGTGNNNNNNNNNNNNNNNNNNNNNNNNNN

. . . . . G/V . A . . . ---------------------------

*P. depressus*5 GGTGCCCAAGGGATGGkTGGAGCAGTTGGAGGTGNNNNNNNNNNNNNNNNNNNNNNNNNN

..**.*.....*....*.****..*.**.*....**************************

**Sanger**

G A Q G M G G P V G G G/V P M G G P P Q F

*S. purpuratus*1 GGCGCTCAAGGAATGGGTGGACCAGTCGGAGGTGkACCGATGGGTGGACCTCCACAATTT

. . . . . . . . . . . G Q . . R . . . .

*S. droebachiensis*6 GGTGCTCAAGGAATGGGTGGACCAGTCGGTGGTGGACAGATGGGTAGACCTCCACAATTT

. . . . . . . . . . . G Q . . R . . . .

*S. droebachiensis*7 GGTGCTCAAGGAATGGGTGGACCAGTCGGTGGTGGACAGATGGGTAGACCTCCACAATTT

. . . . . . R . . . . G . . . . . . . .

*S. pallidus*1 GGCGCTCAAGGAATGGGTAGACCAGTCGGAGGTGGACCGATGGGTGGACCTCCACAATTT

. . . . . . R . . . . G . . . . . . . .

*S. pallidus*3 GGCGCTCAAGGAATGGGTAGACCAGTCGGAGGTGGACCGATGGGTGGACCTCCACAATTT

. . . . . . . A F G/R . G Q G M G G A V R

*S. franciscanus*2 GGCGCCCAAGGGATGGGTGGAGCATTTrGAGGTGGTCAAGGAATGGGTGGAGCAGTCAGA

. T . . . . . A . . . G Q G M G G A V G

*S. nudus*1 GGCACCCAAGGGATGGGTGGGGCAGTTGGAGGTGGTCAAGGGATGGGTGGAGCAGTCGGA

. . . . . G/V . A . . . G Q G M G G A F G

*P. depressus*5 GGTGCCCAAGGGATGGkTGGAGCAGTTGGAGGTGGTCAAGGGATGGGTGGAGCATTTGGA

..**.*.....*..*.*...**..*.*..*..*..*************************

Figure S24. Comparison of Illumina and Sanger alignment data for a region in exon 4 of SPU_014051 (*Sp-Bindin*). Amino acids removed prior to PAML tests are underlined in the Illumina alignment. The gap in the Illumina alignment for the last 9 amino acids is caused by different repeating motifs that are unaligned in the Sanger data. The presence of different amino acid repeats across this region resulted in missing data but no incorrect insertions or deletions. The region represents amino acids 136–155 of SPU_014051.

Figure S25. Patterns of positive selection and constraint for molecular function GO terms. Plotted are the distributions of the mean *d*_N_/*d*_S_ ratio and the negative log10 of the probability of positive selection for each GO term. The dashed lines represent the threshold for positive selection. Note that most GO categories have mean *d*_N_/*d*_S_ ratios below 0.20 indicating strong purifying selection.


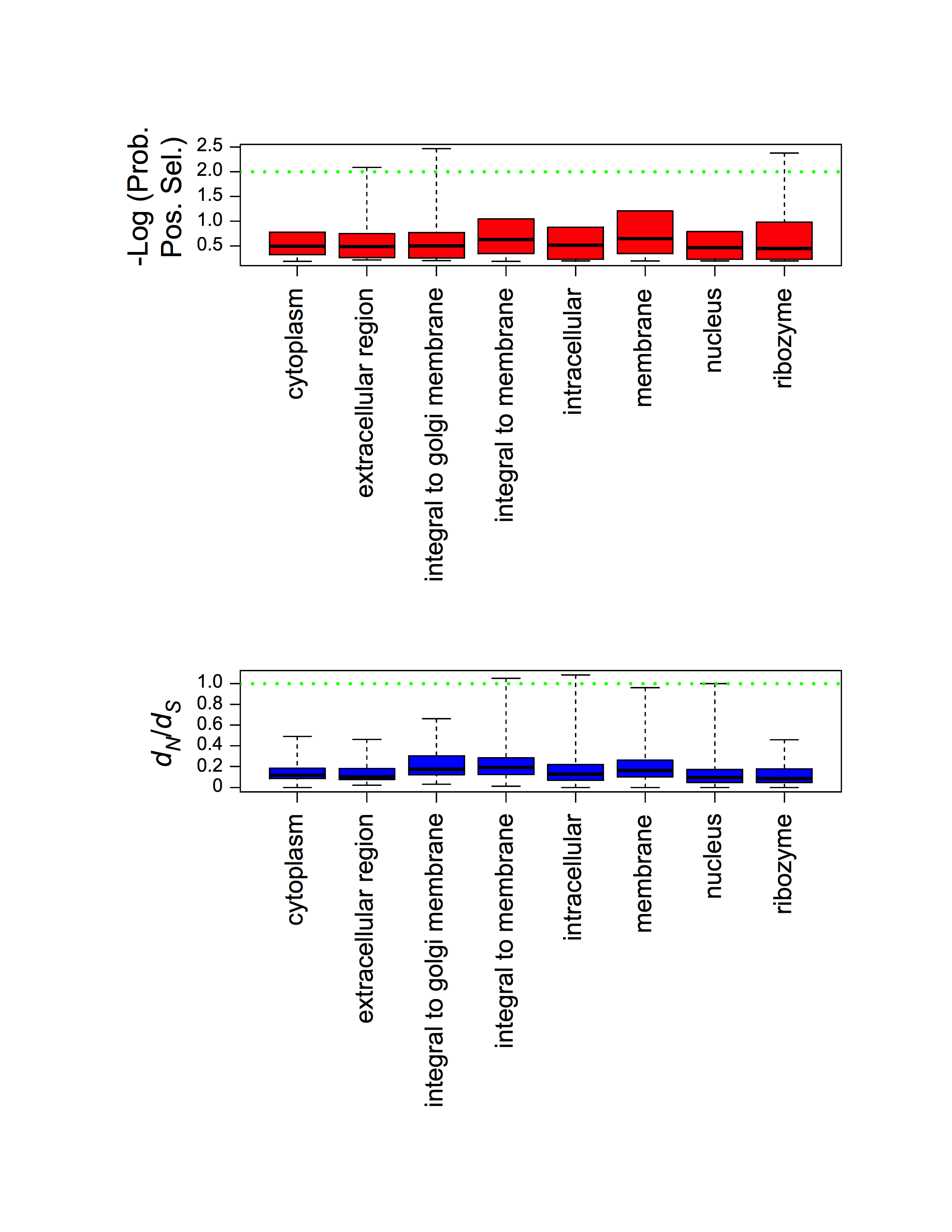


Figure S26. Patterns of positive selection and constraint for cellular component GO terms. Plotted are the distributions of the mean *d*_N_/*d*_S_ ratio and the negative log10 of the probability of positive selection for each GO term. The dashed lines represent the threshold for positive selection. Note that most GO categories have mean *d*_N_/*d*_S_ ratios below 0.20 indicating strong purifying selection.

Figure S27. Patterns of positive selection and constraint for biological process GO terms. Plotted are the distributions of the mean *d*_N_/*d*_S_ ratio and the negative log10 of the probability of positive selection for each GO term. The dashed lines represent the threshold for positive selection. Note that most GO categories have mean *d*_N_/*d*_S_ ratios below 0.20 indicating strong purifying selection.
